# Cognitive and Developmental Functions in Autistic and Non-Autistic Children and Adolescents: Evidence from the Intelligence and Development Scales–2

**DOI:** 10.3390/jintelligence10040112

**Published:** 2022-11-21

**Authors:** Salome D. Odermatt, Wenke Möhring, Silvia Grieder, Alexander Grob

**Affiliations:** 1 Department of Psychology, University of Basel, 4055 Basel, Switzerland; 2 Department of Educational Psychology and Health Psychology, University of Education Schwäbisch Gmünd, 73525 Schwäbisch Gmünd, Germany

**Keywords:** autism spectrum disorder, cognitive functions, developmental functions, Intelligence and Development Scales–2, children and adolescents

## Abstract

Autistic individuals often show impairments in cognitive and developmental domains beyond the core symptoms of lower social communication skills and restricted repetitive behaviors. Consequently, the assessment of cognitive and developmental functions constitutes an essential part of the diagnostic evaluation. Yet, evidence on differential validity from intelligence and developmental tests, which are commonly used with autistic individuals, varies widely. In the current study, we investigated the cognitive (i.e., intelligence, executive functions) and developmental (i.e., psychomotor skills, social–emotional skills, basic skills, motivation and attitude, participation during testing) functions of autistic and non-autistic children and adolescents using the Intelligence and Development Scales–2 (IDS-2). We compared 43 autistic (*M*_age_ = 12.30 years) with 43 non-autistic (*M*_age_ = 12.51 years) participants who were matched for age, sex, and maternal education. Autistic participants showed significantly lower mean values in psychomotor skills, language skills, and the evaluation of participation during testing of the developmental functions compared to the control sample. Our findings highlight that autistic individuals show impairments particularly in motor and language skills using the IDS-2, which therefore merit consideration in autism treatment in addition to the core symptoms and the individuals’ intellectual functioning. Moreover, our findings indicate that particularly motor skills might be rather neglected in autism diagnosis and may be worthy of receiving more attention. Nonsignificant group differences in social–emotional skills could have been due to compensatory effects of average cognitive abilities in our autistic sample.

## 1. Introduction

Autism spectrum disorder (ASD) is a neurodevelopmental disorder characterized by difficulties in social communication and interaction accompanied by restricted repetitive behaviors, activities, and interests (American Psychiatric Association 2013). The worldwide prevalence of ASD has increased in recent years to approximately 1–2% (Idring et al. 2015; Maenner et al. 2020) and ASD is now considered a comparatively frequent condition (Happé and Frith 2020). Autistic individuals often experience difficulties beyond the core symptoms, such as impairments in cognitive and developmental domains, which in turn predict long-term development (e.g., Howlin and Moss 2012). Information about each individual’s cognitive and developmental abilities is particularly important when it comes to making decisions about access to social services, the selection of appropriate treatment programs, and educational placement (White et al. 2007). Moreover, the amount of provided support is oftentimes determined on the basis of a cognitive assessment (Bowen 2014). According to the criteria of the *Diagnostic and Statistical Manual of Mental Disorders* (5th ed.; American Psychiatric Association 2013) and the *International Statistical Classification of Diseases and Related Health Problems* (11th ed.; World Health Organization 2018), clinicians have to report potential difficulties such as intellectual and language impairments in the diagnostic evaluation. Therefore, assessments with intelligence and developmental test batteries—in addition to autism-specific test procedures—represent a core part of the diagnostic process for autistic children and adolescents.

Yet, current tests for children and adolescents mainly allow the assessment of only single characteristics, such as intelligence, at a time and test batteries including multiple cognitive and developmental functions are missing so far. Consequently, when information about several domains or a broad assessment in a diagnostic evaluation is needed, clinicians often have to use various tests. This can be challenging, as the theoretical background and test administration differ widely among tests and dealing with these differences requires resources from the clinician. Moreover, tests build upon different characteristics of standardization samples and thus show less comparable scaled scores. The Intelligence and Development Scales–2 (IDS-2; Grob and Hagmann-von Arx 2018a) is a standardized test battery that assesses cognitive (i.e., intelligence and executive functions) and developmental (i.e., psychomotor skills, social–emotional skills, basic skills, motivation and attitude, and participation during testing) functions in 5- to 20-year-olds. The IDS-2 thus provides a comprehensive picture of an individual’s strengths and difficulties with a single test battery across a wide age range from childhood to adolescence. In addition, the IDS-2 contains clear instructions and structured tasks, and many subtests use a closed-response format, which is particularly important for autistic children because of frequent structural language difficulties (Boucher 2012), making it suitable for administration with autistic individuals. Since the publication of the IDS-2 in 2018, it has often been used in psychological and medical practice in German-speaking countries. Further international adaptations for several other languages are currently in progress or have recently been published (e.g., Dutch, English, Italian, Polish; Grob et al. 2018, 2019, 2021, 2022). In the present study, we aimed to compare autistic children and adolescents to a matched non-autistic control sample on cognitive and developmental functions to study the differential validity of test scores from the IDS-2. By doing so, we can assess whether the IDS-2 is able to distinguish between clinical subgroups and typically developing individuals (Schmidt-Atzert and Amelang 2012).

Although general intellectual functioning varies substantially among autistic individuals, the latest report from the Centers for Disease Control and Prevention showed that almost 60% of autistic children are classified in the below-average intelligence range (IQ < 85), with about half of these children meeting criteria for intellectual disability (IQ ≤ 70; Maenner et al. 2020). Autistic individuals typically display uneven cognitive profiles, with relative strengths in nonverbal domains (e.g., Coolican et al. 2008; Grondhuis et al. 2018) and in tasks assessing abstract reasoning and visuospatial abilities (Charman et al. 2011; Nader et al. 2016), such as a well-documented peak in the Block Design subtest of the Wechsler Intelligence Scales (e.g., Muth et al. 2014). In contrast, relative weaknesses have been demonstrated in verbal domains, particularly in the Comprehension subtest of the Wechsler Intelligence Scales (e.g., Oliveras-Rentas et al. 2012), and in processing speed and working memory tasks^1^ (Mayes and Calhoun 2003a; Nader et al. 2016; Oliveras-Rentas et al. 2012).

Autistic individuals often experience further cognitive difficulties on measures assessing executive functions (e.g., Hill 2004). Executive functions include a set of mental top-down regulation and control mechanisms (Miyake and Friedman 2012). In the theory of executive dysfunction, it is assumed that impairments in executive functions are responsible for some of the autism symptoms (Pennington and Ozonoff 1996), such as repetitive behavior (e.g., de Vries and Geurts 2012; Yerys et al. 2009). Demetriou et al. (2018) reported in the largest meta-analysis to date (235 studies) that autistic individuals showed moderate impairments in executive functions, both overall and in subdomains such as cognitive flexibility, fluency, planning, and inhibition,—which are also assessed with the IDS-2 (see Table S1 in the Supplement for an overview)—compared to non-autistic individuals. 

Moreover, previous research showed significant impairments in autistic individuals’ motor abilities, beginning in early childhood with deficits in the acquisition of motor milestones, such as later independent walking (e.g., Manicolo et al. 2019), and delays in gross and fine motor skills, for example, diminished object manipulation activity (Libertus et al. 2014; Provost et al. 2007). In a recent meta-analysis of 139 studies with samples of autistic children, adolescents, and young adults, their overall motor ability as well as gross and fine motor skills were strongly impaired in comparison to non-autistic peers (Coll et al. 2020). In line with this result, several studies found that autistic children, compared to non-autistic samples, scored lower on subscales (i.e., manual dexterity, ball skills, and balance) of the Movement Assessment Battery for Children–2 (M-ABC-2; Petermann 2008), which is a test of motor development that contains tasks similar to those in the IDS-2 psychomotor skills domain (Liu and Breslin 2013; Manicolo et al. 2019; Siaperas et al. 2012).

Further, research has shown that lower motor skills of autistic children were significantly associated with poorer social communication skills (MacDonald et al. 2013b). It has been suggested that motor problems might even precede social and communication deficits in autistic individuals because they may limit social participation and interaction with peers during play and may interfere with effective and timely movements, such as turning the head or pointing to something, that are particularly important for joint attention (Bhat et al. 2011). Impairments in social communication and interaction, such as difficulties in social–emotional reciprocity and nonverbal communicative behaviors, as well as in developing, maintaining, and understanding relationships constitute a core diagnostic characteristic of ASD (American Psychiatric Association 2013; World Health Organization 2018). These impairments are reflected in less accurate emotion recognition in human faces, with increased response times (Leung et al. 2022; Yeung 2022), more maladaptive emotion regulation strategies (Cai et al. 2018), including more reliance on others to regulate their emotions (Cibralic et al. 2019), and fewer socially competent behaviors (e.g., Meyer et al. 2009) compared to non-autistic individuals.

Additionally, language difficulties commonly co-occur with autism (Kjellmer et al. 2018). Some autistic individuals do not acquire verbal language at all (Brignell et al. 2018). Among those who develop language, delays often begin in infancy with retardations in the production of first words and in early language comprehension (e.g., Luyster et al. 2007; Mitchell et al. 2006). Moreover, across the preschool years, autistic children exhibit difficulties in phonological awareness skills (e.g., identifying syllables or onset-rimes), with slower development than their non-autistic peers (Dynia et al. 2019). Regarding language production and comprehension (i.e., expressive and receptive language skills, respectively), some studies indicated an atypical pattern, with better expressive and poorer receptive language skills in autistic individuals (e.g., Hudry et al. 2010). However, a meta-analysis examining 74 studies reported that autistic children and adolescents had scores that were approximately 1.5 standard deviations lower in receptive *as well as* expressive language abilities compared to non-autistic samples (Kwok et al. 2015).

In terms of academic skills, research indicated that autistic students demonstrate variable performance (Keen et al. 2016). Specifically, in previous studies, autistic individuals showed similar basic word-reading skills, such as word recognition, compared to non-autistic peers, but they tended to have difficulties in reading comprehension (for a meta-analysis: Brown et al. 2013). Autistic individuals with higher (vs. lower) reading skills also seemed to demonstrate better writing abilities (Zajic et al. 2020). Studies predominantly indicated deficits in text generation abilities for autistic individuals, while overall intact or slightly impaired spelling skills were reported (Finnegan and Accardo 2018; Mayes and Calhoun 2003a, 2003b). Similarly, the majority of autistic individuals exhibited average competencies in mathematics, such as mathematical problem solving, compared to non-autistic peers or to the norm population in previous research (Chiang and Lin 2007; Titeca et al. 2017; Troyb et al. 2014).

Concerning motivation and attitude, a recent meta-analysis reported that autistic individuals displayed significantly lower levels of conscientiousness than non-autistic individuals (Lodi-Smith et al. 2019). In contrast, less is known regarding achievement motivation in autistic individuals. A few studies reported that autistic individuals encountered problems with self-regulation (e.g., Jahromi et al. 2012; Konstantareas and Stewart 2006) and displayed higher interest in mathematics while simultaneously showing more fear of failure and lower mastery goals (Georgiou et al. 2018). Moreover, autistic children tended to exhibit impaired engagement (Keen 2009), especially in assessment situations where they frequently demonstrated off-task behaviors (Akshoomoff 2006) and a lack of willingness to complete tasks (Mandelbaum et al. 2006).

Previous research has rarely used the IDS-2 in order to test autistic individuals. The only study so far reported in the technical manual of the IDS-2 (Grob and Hagmann-von Arx 2018b) built upon a small sample of autistic children and adolescents (*N* = 18; *M*_age_ = 13 years 4 months, age range 8–17 years; 17 males and 1 female). Findings showed significantly lower group mean values for autistic children and adolescents compared to non-autistic peers in the composite score of social–emotional skills (*d* = 0.62) and the composite score of psychomotor skills (*d* = 1.01) of the IDS-2. No differences were found in the composite scores of other domains. However, evidence of possible differences at the level of subtests is currently lacking, as analyses on this level have not been performed. Moreover, the study included mainly children and adolescents with Asperger’s syndrome (*n* = 13) and no participants with previously diagnosed infantile autism. Given the small sample size, which may have diminished the power to find group differences, and the biased distribution of sex and subtype, it remains unknown to what extent these results can be generalized.

Building on this theoretical background, we pursued two goals for the present study: First, we aimed to extend previous research on various cognitive and developmental functions in autistic children and adolescents using a single test procedure and based on the norms of a large and representative standardization sample. By doing so, our findings will provide a comparable and comprehensive view of participants’ performance in relevant domains. Second, we aimed to add knowledge regarding the differential validity evidence for test scores of the IDS-2 in autistic individuals, as psychological test procedures need to be examined in terms of their scientific quality in order to draw appropriate conclusions based on their test results. Given that previous research had some limitations (Grob and Hagmann-von Arx 2018b), we attempted to overcome these shortcomings by assessing a larger sample, including a more representative mapping of sex and subtypes, and performing analyses at the level of subtests, which have not yet been investigated in this population. We therefore examined possible mean-level differences between a large sample of autistic children and adolescents and a control sample of non-autistic children and adolescents matched by age, sex, and maternal education in the cognitive and developmental functions measured by the IDS-2. We included maternal education as a proxy for socioeconomic status (SES) to control for the fact that more autistic children and adolescents come from families with higher SES than from other SES groups (Thomas et al. 2012; Van Meter et al. 2010). 

Taking into consideration the presented literature, we hypothesized that autistic children and adolescents would score lower than the control sample of non-autistic children in the following IDS-2 domains as displayed in Table 1, while we assumed that autistic children and adolescents’ scores would be similar to those of the control sample in the other IDS-2 domains (see Table 1 for a summary).

## 2. Materials and Methods

### 2.1. Participants and Procedure

Forty-three autistic children and adolescents (*M*_age_ = 12 years 4 months, age range 7–17 years; 35 males and 8 females) were recruited during (*n* = 18) or after (*n* = 25) the IDS-2 standardization and validation study with the help of local child and adolescent psychiatric services and hospitals, privately practicing psychiatrists and psychotherapists who are experts in autism diagnoses, and associations for autistic individuals. All included children and adolescents were diagnosed with ASD (infantile autism: *n* = 11, atypical autism: *n* = 6, Asperger’s syndrome: *n* = 24, not specified: *n* = 2) but were not selected on the basis of specific subtypes. Participants had received the diagnosis on average 4.08 years (*SD* = 2.61) prior to their participation in the present study. The ratio of males to females corresponded to the distribution of approximately four males to one female diagnosed with ASD in the population (Maenner et al. 2020).

A control sample of 43 non-autistic children and adolescents (*M*_age_ = 12 years 6 months, age range 6–20 years; 35 males and 8 females) was drawn from the German standardization and validation sample of the IDS-2 (N = 2030; Mage = 12 years 3 months, age range 5–20 years; 977 males and 1053 females). The control sample was matched by age, sex, and maternal education (as a proxy for SES) and did not differ regarding demographic characteristics from the sample of autistic children and adolescents (see Table 2). Non-autistic children and adolescents were recruited from kindergartens and schools.

All participants were individually tested using the IDS-2 by psychologists or trained psychology students. For the administration of the IDS-2 with autistic children and adolescents, we received input from psychiatrists and psychotherapists who specialize in autism. Test administration lasted approximately 4 h and was split into two sessions no longer than 1 week apart upon a participant’s request. Participants were tested either at their homes or in a laboratory at the university. The local ethics committee (Ethics Committee Northwest and Central Switzerland) provided approval and the study was conducted in accordance with the Declaration of Helsinki. Written informed consent was obtained from participants and/or their parents.

### 2.2. Instrument

A detailed description of the IDS-2 (Grob and Hagmann-von Arx 2018a) can be found in the Supplemental Material (Table S1). Psychometric properties have been demonstrated in several studies for the standardization sample (Grieder and Grob 2020; Grob and Hagmann-von Arx 2018b). Demographic characteristics were assessed through a parental interview at the beginning of the first test session.

### 2.3. Statistical Analyses

Analyses were conducted with R (R Core Team 2021). To obtain a non-autistic sample that would be comparable to the autistic sample with respect to demographic characteristics, we performed a matching procedure using the MatchIt package (Ho et al. 2011). We matched the two samples by age (nearest; continuous), sex (exact; 0 = male, 1 = female), and maternal education (nearest; 1 = compulsory school, 2 = apprenticeship, 3 = high school, 4 = higher vocational education, 5 = university degree, 6 = other, 7 = unknown). We calculated independent-samples *t* tests to investigate mean-level differences between the autistic sample and the non-autistic sample in cognitive and developmental domains using standardized scores (*M* = 100, *SD* = 15, for Profile IQ, Full-Scale IQ, Screening IQ, and the seven intelligence group factors; *M* = 10, *SD* = 3, for other composite scores and subtests). To reduce the alpha error inflation caused by multiple testing, *p* values were adjusted with Hommel’s (1988) correction by including *p* values from all tests simultaneously. Effect sizes were computed (Cohen 1988) and interpreted in accordance with common practice (Cohen’s *d*; small effect: *d* ≥ 0.20, medium effect: *d* ≥ 0.50, large effect: *d* ≥ 0.80). A post hoc power analysis using G*Power (Faul et al. 2007) revealed that with α = .05 and power = .80, small effects (*d* = 0.30) could be detected in the present sample (note that this is without accounting for multiple testing). Differences were interpreted as meaningful if they were significant after Hommel’s correction and showed at least a small effect size. In addition, we reported reliabilities for all IDS-2 scores, consisting of Cronbach’s alpha for homogeneous subtests; reliabilities calculated according to a formula of Lienert and Raatz (1998) for composite scores, which are based on intercorrelations and reliabilities of those subtests or tasks that are included in the corresponding score; or retest reliabilities reported in the technical manual of the IDS-2 (Grob and Hagmann-von Arx 2018b) for subtests that contain a single score or consist of heterogeneous tasks.

## 3. Results

Reliabilities, descriptive statistics, and results of the independent-samples *t* tests^2^ are presented in Table 3 for the cognitive functions and in Table 4 for the developmental functions. Reliabilities were high for composite scores and high-to-satisfactory for subtests in both samples.

### 3.1. Cognitive Functions

**Figure 1:**
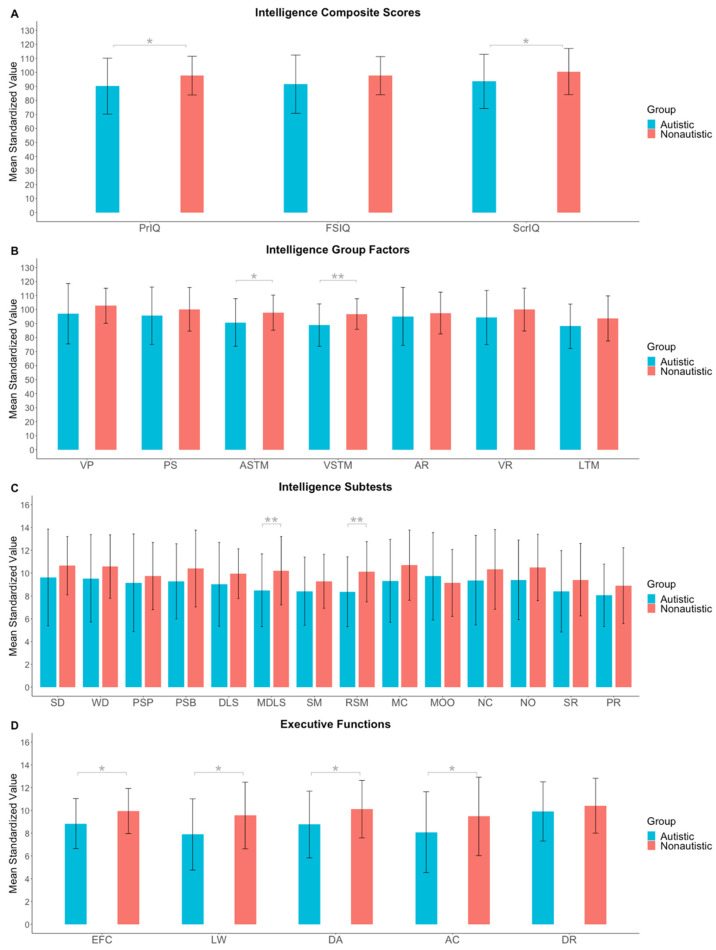
Means and standard deviations are reported for (**A**) intelligence composite scores, (**B**) intelligence group factors, (**C**) intelligence subtests, and (**D**) executive functions composite score and subtests of the Intelligence and Development Scales–2 for autistic and non-autistic children and adolescents. Asterisks in grey indicate *p* values not adjusted with Hommel’s (1988) correction. Asterisks in black indicate *p* values adjusted according to Hommel (1988). Please note that after this correction, none of the comparisons were significant and therefore, no black asterisks are included in the present graphs. PrIQ = Profile IQ; FSIQ = Full-Scale IQ; ScrIQ = Screening IQ; VP = Visual Processing; PS = Processing Speed; ASTM = Auditory Short-Term Memory; VSTM = Visuospatial Short-Term Memory; AR = Abstract Reasoning; VR = Verbal Reasoning; LTM = Long-Term Memory; SD = Shape Design; WD = Washer Design; PSP = Parrots; PSB = Boxes; DLS = Digit and Letter Span; MDLS = Mixed Digit and Letter Span; SM = Shape Memory; RSM = Rotated Shape Memory; MC = Matrices: Completion; MOO = Matrices: Odd One Out; NC = Naming Categories; NO = Naming Opposites; SR = Story Recall; PR = Picture Recall; EFC = Executive functions composite score; LW = Listing Words; DA = Divided Attention; AC = Animal Colors; DR = Drawing Routes. * *p* < .05. ** *p* < .01. *** *p* < .001.

Figure 1 displays the means and standard deviations in the cognitive functions of the IDS-2 for the autistic and non-autistic samples. Before controlling for multiple testing, we found significant group differences for the intelligence composite scores: Profile IQ, *t*(77) = 1.96, *p* = .027, and Screening IQ, *t*(82) = 1.80, *p* = .038, with small effect sizes (*d* = 0.44 and 0.39, respectively), indicating lower scores for the autistic sample than the control sample. Furthermore, we observed group differences for the intelligence group factors: Auditory Short-Term Memory, *t*(79) = 2.12, *p* = .019, and Visuospatial Short-Term Memory, *t*(79) = 2.70, *p* = .004, with small-to-medium effect sizes (*d* = 0.47 and 0.60, respectively), and the corresponding subtests Mixed Digit and Letter Span, *t*(79) = 2.51, *p* = .007, and Rotated Shape Memory, *t*(79) = 2.78, *p* = .003, with medium effect sizes (*d* = 0.56 and 0.62, respectively), such that the autistic participants showed lower mean values than the control sample. Moreover, the autistic participants had significantly lower mean values in the executive functions composite score, *t*(71) = 2.27, *p* = .013, and the subtests Listing Words, *t*(73) = 2.38, *p* = .010, Divided Attention, *t*(71) = 2.13, *p* = .019, and Animal Colors, *t*(72) = 1.70, *p* = .047. Effect sizes were in the small-to-medium range (*d* = 0.40 to 0.55). We found no differences between autistic and non-autistic participants in the Full-Scale IQ, *t*(81) = 1.58, *p* = .059, in the intelligence group factors Visual Processing, *t*(80) = 1.46, *p* = .148, Processing Speed, *t*(80) = 1.15, *p* = .126, Abstract Reasoning, *t*(80) = 0.62, *p* = .539, Verbal Reasoning, *t*(81) = 1.48, *p* = .071, and Long-Term Memory, *t*(79) = 1.57, *p* = .060, including corresponding intelligence subtests, and in the executive functions subtest Drawing Routes, *t*(74) = 0.88, *p* = .192.

However, after controlling for multiple testing, the significant differences in intelligence and executive functions fell above the Hommel-corrected *p*-value threshold (see Table 3).

### 3.2. Developmental Functions

**Figure 2:**
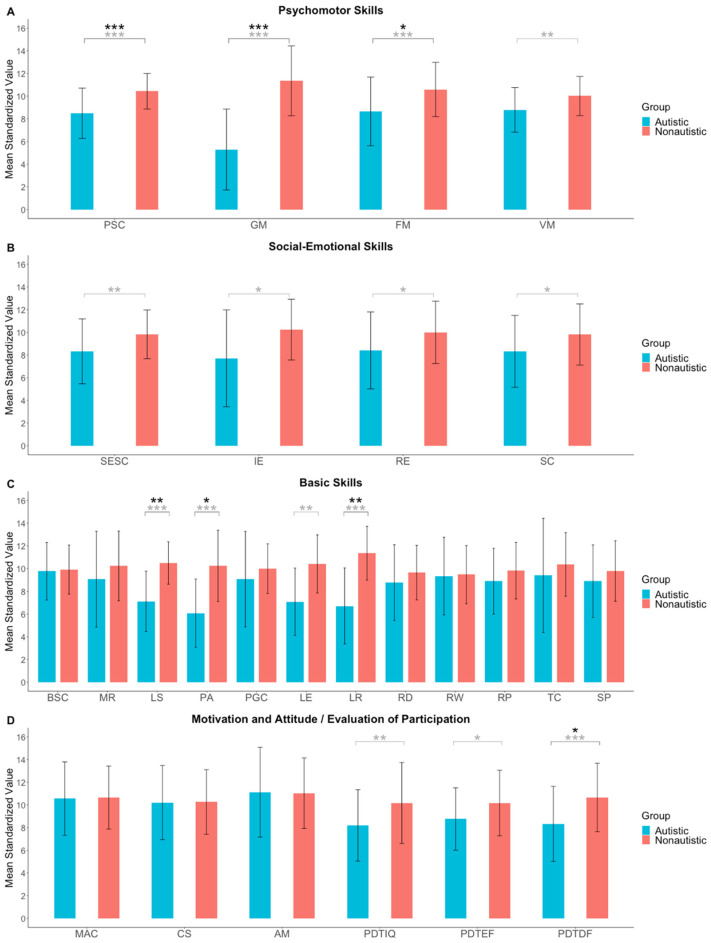
Means and standard deviations are reported for (**A**) psychomotor skills composite score and subtests, (**B**) social–emotional skills composite score and subtests, (**C**) basic skills composite score and subtests, and (**D**) motivation and attitude composite score and subtests as well as for the evaluation of participation during testing of the Intelligence and Development Scales–2 for autistic and non-autistic children and adolescents. Asterisks in grey indicate *p* values not adjusted with Hommel’s (1988) correction. Asterisks in black indicate *p* values adjusted according to Hommel (1988). PSC = Psychomotor skills composite score; GM = Gross Motor Skills; FM = Fine Motor Skills; VM = Visuomotor Skills; SESC = Social–emotional skills composite score; IE = Identifying Emotions; RE = Regulating Emotions; SC = Socially Competent Behavior; BSC = Basic skills composite score; MR = Logical–Mathematical Reasoning; LS = Language Skills; PA = Phoneme Analysis; PGC = Phoneme–Grapheme Correspondence; LE = Language Expressive; LR = Language Receptive; RD = Reading; RW = Reading Words; RP = Reading Pseudo Words; TC = Text Comprehension; SP = Spelling; MAC = Motivation and attitude composite score; CS = Conscientiousness; AM = Achievement Motivation; PDTIQ = Participation during testing, intelligence; PDTEF = Participation during testing, executive functions; PDTDF = Participation during testing, developmental functions. * *p* < .05. ** *p* < .01. *** *p* < .001.

Figure 2 shows the means and standard deviations in the developmental functions of the IDS-2 for the autistic and non-autistic samples. Before controlling for multiple testing, results indicate that autistic participants scored significantly lower than non-autistic participants in psychomotor skills [composite score, *t*(81) = 4.60, *p* < .001; Gross Motor Skills, *t*(32) = 5.30, *p* < .001; Fine Motor Skills, *t*(79) = 3.20, *p* < .001; Visuomotor Skills, *t*(81) = 3.01, *p* = .002] with medium-to-large effect sizes (*d* = 0.66 to 1.82). We found a similar group difference for participants’ social–emotional skills [composite score, *t*(82) = 2.71, *p* = .004; Identifying Emotions, *t*(32) = 2.07, *p* = .023; Regulating Emotions, *t*(82) = 2.37, *p* = .010; Socially Competent Behavior, *t*(80) = 2.29, *p* = .012] with medium effect sizes (*d* = 0.51 to 0.71), and in language skills [composite score, *t*(28) = 4.11, *p* < .001; Phoneme Analysis, *t*(29) = 3.75, *p* < .001; Language Expressive, *t*(28) = 3.31, *p* = .001; Language Receptive, *t*(29) = 4.52, *p* < .001] with large effect sizes (*d* = 1.22 to 1.63). Furthermore, autistic participants showed significantly lower group mean values than the control sample for the evaluation of participation during the test session of intelligence, *t*(81) = 2.68, *p* = .004, executive functions, *t*(71) = 2.13, *p* = .018, and developmental functions, *t*(79) = 3.30, *p* < .001, with medium effect sizes (*d* = 0.50 to 0.73). We found no differences in the subtests Logical–Mathematical Reasoning, *t*(81) = 1.44, *p* = .153, Reading, *t*(74) = 1.35, *p* = .182, Spelling, *t*(65) = 1.26, *p* = .212, and in the motivation and attitude domain [composite score, *t*(46) = 0.11, *p* = .458; Conscientiousness, *t*(45) = 0.06, *p* = .477; Achievement Motivation, *t*(47) = −0.07, *p* = .528], indicating similar performance in autistic and non-autistic participants.

After controlling for multiple testing, significant group differences remained for the composite score of psychomotor skills (*p*_H_ < .001) and subtests Gross Motor Skills (*p*_H_ < .001) and Fine Motor Skills (*p*_H_ = .046). Moreover, the composite score of language skills remained significant (*p*_H_ = .008) as well as Phoneme Analysis (*p*_H_ = .019) and Language Receptive (*p*_H_ = .003) tasks. Finally, the evaluation of participation during testing of the developmental functions remained significant (*p*_H_ = .035; see Table 4).^3^

### 3.3. Post Hoc Analyses

To assess for age-related differences between children and adolescents, we further performed post hoc analyses separately for children aged 5–10 years (*n* = 17) and adolescents aged 11–20 years (*n* = 26). After Hommel’s (1988) correction, autistic children scored significantly lower than non-autistic children in the composite scores of the cognitive functions, the intelligence group factors, Auditory Short-Term Memory, Visuospatial Short-Term Memory, and Verbal Reasoning (including the corresponding subtests) as well as in psychomotor skills, social–emotional skills, and basic skills of the developmental functions (see Tables S3 and S4 in the Supplemental Material for results). We found no significant group differences between autistic and non-autistic adolescents for the cognitive and developmental functions of the IDS-2 after controlling for multiple testing (see Tables S5 and S6 in the Supplemental Material).

## 4. Discussion

In the present study, we compared autistic children and adolescents to a matched control sample on six cognitive and developmental functions assessed with the IDS-2. Our results provide evidence for differential validity for the IDS-2 test scores in psychomotor skills, language skills, and in the evaluation of participation during testing of the developmental functions, with autistic children and adolescents scoring lower than non-autistic participants in these domains. No group differences were detected in the other domains after controlling for multiple testing. Overall, our findings provide an overview of important cognitive and developmental functions in autistic children and adolescents using a single comprehensive and standardized test battery.

In line with our hypotheses, we found similar performance in autistic and non-autistic participants for the intelligence group factors Visual Processing and Abstract Reasoning, which corresponds to studies reporting relative strengths for autistic individuals in nonverbal domains (e.g., Grondhuis et al. 2018) and in subtests measuring fluid reasoning and visuospatial abilities (Charman et al. 2011; Nader et al. 2016). Specifically, the Shape Design subtest, which is part of the Visual Processing group factor of the IDS-2, requires participants to reproduce presented geometric figures with rectangles and triangles. This task is similar to the Block Design subtest of the Wechsler Intelligence Scales, for which autistic individuals oftentimes show at least comparable performance to non-autistic controls (e.g., Muth et al. 2014).

However, in contrast to our hypotheses and previous research (e.g., Demetriou et al. 2018), no significant group differences emerged for the other cognitive functions scores of the IDS-2 after correcting for multiple testing, even though effect sizes were in the small-to-medium range. This finding suggests that our autistic sample included participants with overall average cognitive abilities. One explanation for this result could be that about half of our autistic participants had been diagnosed with Asperger’s syndrome, which is known for impairments in social interaction and restricted interests, but without deficits in cognitive development (10th ed.; World Health Organization 2016). Moreover, when assessing age-related differences in a set of post hoc analyses, we found that autistic adolescents scored similarly to non-autistic adolescents in the IDS-2, while autistic children obtained significantly lower scores in several domains of the IDS-2 compared to non-autistic children. In particular, group differences between autistic and non-autistic children remained significant after controlling for multiple testing in the composite scores of the intelligence and executive functions domains as well as in the intelligence group factors Verbal Reasoning and Auditory and Visuospatial Short-Term Memory. These results are in line with previous research reporting weaknesses of autistic children in verbal domains (e.g., Oliveras-Rentas et al. 2012) and in working memory tasks (e.g., Mayes and Calhoun 2003a) as the IDS-2 Auditory and Visuospatial Short-Term Memory group factors also include tasks measuring working memory (i.e., [Mixed] Digit and Letter Span—backwards and Rotated Shape Memory; see Table S1 in the Supplement). In addition, autistic children scored lower on motor and language skills, and importantly, also on social–emotional skills. Interestingly, we did not find any differences between autistic and non-autistic participants when focusing on adolescents only. One reason for this finding could be that autistic adolescents have already received support and intervention in crucial developmental areas, whereas the included autistic children may have been recently diagnosed with autism and thus have had little or no treatment to that point. However, it should be noted that these results are based on small sample sizes. Thus, future studies should use larger age-specific samples to investigate developmental effects across childhood and adolescence and simultaneously control for previous interventions.

Autistic participants had significant impairments in overall psychomotor skills as well as lower scores in gross and fine motor skills in the IDS-2 compared to the non-autistic participants. This finding is in line with results of a previous meta-analysis (Coll et al. 2020) and studies using the M-ABC-2 to assess motor abilities (e.g., Manicolo et al. 2019). Motor skills are particularly important for carrying out everyday tasks (e.g., grasping a glass) and performing activities of daily living (MacDonald et al. 2013a), as well as for participating in activities at school or in the community (Oliveira et al. 2021). It has been suggested that one reason for these motor differences may be that autistic individuals encounter problems in the translation of sensory inputs into movements (Hannant et al. 2016). Moreover, structural and functional alterations in motor cortex regions of the brain (Mostofsky et al. 2007; Nebel et al. 2014) and in the cerebellum (Fatemi et al. 2012; Mostofsky et al. 2009) have been detected for autistic individuals, which might explain some of the motor impairments. The strong group difference we observed in gross motor skills, representing the largest effect in our study, is in accordance with previous research (Coll et al. 2020) and may be associated with the high prevalence of autistic individuals exhibiting hypotonia (51%) or motor apraxia (34%; Ming et al. 2007). Hence, autistic individuals tend to experience difficulties especially in movements that require activation of muscles in the entire body including balance, arm movements, and coordination. However, as this subtest is administered only to 5- to 10-year-olds in the IDS-2 and correlational research has shown that autistic children’s motor skills improve with age (Coll et al. 2020), future longitudinal studies are needed to study possible developmental effects. Although it is not compulsory to report potential difficulties in motor skills as part of the diagnostic criteria of ASD, our findings support the importance of assessing psychomotor abilities during the diagnostic evaluation of children and adolescents at increased likelihood of ASD, as they might be crucial for treatment programs (Bhat et al. 2011; Colombo-Dougovito and Block 2019).

As stated in previous studies, we found that autistic children scored lower in language skills, such as in phoneme analysis (Dynia et al. 2019) and receptive language tasks (Kwok et al. 2015), compared to the non-autistic participants. However, we detected no significant group differences after correcting for multiple testing in expressive language tasks. Although a previous meta-analysis showed equally impaired receptive and expressive language skills in autistic individuals (Kwok et al. 2015), our finding is in line with other studies that also indicated an atypical language pattern of autistic individuals with an advantage in expressive over receptive language skills (e.g., Hudry et al. 2010). One reason for this result might be that we used a direct measurement of language skills in our study. Previous research also found this pattern when using a similar test procedure but did not detect any expressive language advantages when using caregiver reports (Ellis Weismer et al. 2010). Given that having better language production than comprehension skills is contrary to what is generally anticipated in typically developing peers, researchers even suggested that this pattern may be unique to autism (e.g., Volden et al. 2011) and therefore could be used for differential diagnosis (Mitchell et al. 2011) and specific interventions (Hudry et al. 2010). Nevertheless, as the expressive and receptive language tasks are conducted only with 5- to 10-year-olds in the IDS-2 and previous studies have reported a decrease in the expressive–receptive discrepancy in older autistic individuals (Kwok et al. 2015; Volden et al. 2011), it could also be that our result was driven by age effects. Because of the diagnostic and therapeutic potential of this finding, future studies should continue to examine this potential discrepancy between expressive and receptive language in autistic individuals across development.

Additionally, we found no significant group differences in tasks measuring phoneme–grapheme correspondence, which is consistent with our finding that autistic participants also scored similarly to the non-autistic control group in the reading and spelling subtests in our study. This result might be explained by the fact that knowledge of letter–sound correspondence is a prerequisite for the development of literacy skills (Carnine et al. 2010) and therefore needs to be intact for average reading and spelling skills. The finding that our autistic participants showed no differences in the basic skills logical–mathematical reasoning, reading, and spelling compared to non-autistic peers is in line with other studies (e.g., Brown et al. 2013; Chiang and Lin 2007). One reason may refer to the fact that most of the autistic participants in our study attended inclusive educational settings. The enrollment in integrative settings can have a positive impact on autistic individuals’ academic skills as individualized education plans in mainstream programs focus more on academic enhancement than in specialized settings which place more emphasis on life competencies and developmental domains (Kurth and Mastergeorge 2010).

Contrary to previous research (e.g., Cai et al. 2018; Yeung 2022), we found no significant group differences for social–emotional skills after correcting for multiple testing. One explanation for this result could be that the tasks assessing social–emotional skills in the IDS-2 mainly measure explicit knowledge, such as naming socially competent behavior in hypothetical social situations, rather than actual behavior in real-life situations. Since we did not observe any group differences in the cognitive functions of the IDS-2 either, it might be that autistic participants could compensate for difficulties in social–emotional skills with higher-level analytical strategies (Harms et al. 2010; Leung et al. 2022). This would be in line with studies reporting that intelligence is positively associated with social–emotional skills (Jones et al. 2011), especially in autistic individuals (Dyck et al. 2006; Salomone et al. 2019; Trevisan and Birmingham 2016). We found further evidence for this assumption in supplementary analyses where we matched the non-autistic control sample by age, sex, and Full-Scale IQ and obtained lower effect sizes for the social–emotional skills composite score as well as for the subtests Identifying Emotions and Regulating Emotions compared to the effect sizes obtained by matching the samples by age, sex, and maternal education (see Table S2 in the Supplemental Material). In addition, time limits in testing procedures might explain part of the nonsignificant group differences in social–emotional skills. Nagy et al. (2021) found impairments only when time limits for responding were applied, and the present tasks assessing social–emotional skills did not have any time restrictions. However, it is important to note that although meta-analyses and reviews show significant deficits in social–emotional abilities of autistic individuals (e.g., Cai et al. 2018; Yeung 2022), several previous studies were also not able to detect impairments in emotion recognition and regulation (e.g., Jones et al. 2011; Mazefsky et al. 2014; Rosset et al. 2008) or reported difficulties only for certain emotions, for example, for negative emotions (e.g., Shanok et al. 2019). To clarify the interplay between explicit knowledge and social–emotional skills in the IDS-2, future research should use multiple methods to assess social–emotional skills and compare the autistic participants’ performance in the IDS-2 with the behavior they demonstrate in real-life social interactions using observational measures. Even though the group differences in the social–emotional skills of the IDS-2 were no longer significant after correcting for multiple testing, it is crucial to mention that effect sizes were within a medium range and comparable to those in a previous meta-analysis (Yeung 2022) which at least tends to indicate differential validity of test scores from the social–emotional skills domain of the IDS-2.

A strength of our study is that we assessed the cognitive and developmental functions using a standardized test procedure with good psychometric properties. Moreover, we used a single test battery based on one standardization sample for the assessment of a broad range of cognitive and developmental domains. In addition, our sample covered a wide age range and was representative of the autistic population, in that the male:female ratio was approximately 4:1 (Maenner et al. 2020), different subtypes were included, and children and adolescents exhibited known comorbid conditions (Leyfer et al. 2006; Salazar et al. 2015). We also consider it a strength that we included participants with intellectual functioning below 70, which represents an understudied subpopulation in autism research (Russell et al. 2019). In addition, by selecting the control sample through a matching procedure, we could control for possible confounding influences of age, sex, and SES.

The present study also has limitations that need to be considered and addressed in future research. First, we relied on diagnostic evaluations carried out by clinical services and experienced psychiatrists and psychotherapists and hence could not consider the standardization and comparability of the diagnoses. Second, we had no information regarding symptom severity or previous treatment programs and could therefore not control for these factors. Third, analyses were conducted at the group level, which limits generalizability to individuals. Finally, although the sample size was larger than in previous studies, an even larger sample of children and adolescents would further increase the power to detect small effects in future studies.

## 5. Conclusions

In sum, our findings suggest that in particular, motor and language skills as well as achievement motivation rated by the test administrator were impaired in autistic children and adolescents in the IDS-2 compared to non-autistic participants, which provides evidence for differential validity for these domains of the IDS-2. The largest difference was found in gross motor skills. We therefore advise that therapists working with autistic children should gain knowledge in the area of motor and language therapeutic intervention. Speech–language pathologists as well as psychomotor therapists should obtain autism-specific knowledge, so that autistic children with limited motor and language skills receive appropriate therapeutic support regardless of the background of the therapist. Arguably, with optimal training, autistic participants may also perform tasks in the psychomotor and language domains with greater engagement, which, in turn, could have a positive impact on the long-term development of their motor and language abilities. In conclusion, our results highlight important domains beyond the core symptoms of ASD that need to be considered in future research, educational contexts, and clinical assessment and that seem particularly critical for interventions.

## Figures and Tables

**Table 1:** Summary of our hypotheses.

Domain	Assumed Differences in Performance between Autistic and Non-Autistic Participants	Assumed Similar Performance inAutistic and Non-Autistic Participants
	Variable	Variable
Intelligence	Composite scores (Profile IQ, Full-Scale IQ, Screening IQ) Processing Speed Parrots Boxes Auditory Short-Term Memory Digit and Letter Span Mixed Digit and Letter Span Visuospatial Short-Term Memory Shape Memory Rotated Shape Memory Verbal Reasoning Naming Categories Naming Opposites Long-Term Memory Story Recall Picture Recall	Visual Processing Shape Design Washer Design Abstract Reasoning Matrices: Completion Matrices: Odd One Out
Executive functions	Composite score Listing Words Divided Attention Animal Colors Drawing Routes	
Psychomotor skills	Composite score Gross Motor Skills Fine Motor Skills Visuomotor Skills	
Social–emotional skills	Composite score Identifying Emotions Regulating Emotions Socially Competent Behavior	
Basic skills	Language skills Phoneme Analysis Phoneme–Grapheme Correspondence Language Expressive Language Receptive Text Comprehension	Composite score Logical–Mathematical Reasoning Reading Reading Words Reading Pseudo Words Spelling
Motivation and attitude	Composite score Conscientiousness Achievement Motivation	
Participation during testing	intelligence executive functions developmental functions	

*Note.* Differences in performance between autistic and non-autistic participants are interpreted as meaningful if the *p* value is significant after Hommel’s correction and the effect size is at least small.

**Table 2:** Demographic Characteristics of Autistic and Non-Autistic Children and Adolescents.

Characteristic	Autistic Sample *n* = 43		Non-Autistic Sample *n* = 43		χ^2^	*p*
	*n*	%	*n*	%		
Sex					11.33	1.000
Female	8	19	8	19		
Male	35	81	35	81		
Maternal education					14.24	1.000
No postsecondary education	23	54	23	54		
Compulsory school	1	2	2	5		
Apprenticeship	16	37	15	35		
High school	1	2	1	2		
Higher vocational education	5	12	5	12		
Postsecondary education (university degree)	19	44	19	44		
Other	0	0	0	0		
Unknown	1	2	1	2		
Participants’ current education					7.00	1.000
Kindergarten	0	0	1	2		
Elementary school	14	33	20	47		
Secondary school	10	23	11	26		
School for special education	8	19	1	2		
High school	6	14	5	12		
Apprenticeship	3	7	4	9		
University	0	0	1	2		
None	2	5	0	0		
Intelligence level					11.09	1.000
<70	9	21	1	2		
70–84	7	16	6	14		
85–99	8	19	16	37		
100–114	11	26	14	33		
≥115	6	14	6	14		
Comorbid condition					12.15	1.000
Visual impairment	6	14	8	19		
Motor problems	4	9	0	0		
Speech problems	4	9	1	2		
Dyslexia	2	5	2	5		
Dyscalculia	0	0	2	5		
AD(H)D	4	9	4	9		
Depression	1	2	1	2		
Medical problems	10	23	2	5		
Ethnicity					10.04	1.000
German-speaking country	38	88	39	91		
Other European country	4	9	2	5		
Non-European country	1	2	1	2		
Unknown	0	0	1	2		
Native language					10.99	1.000
Monolingual German	32	74	35	81		
Bilingual	6	14	6	14		
Other language than German	5	12	2	5		

*Note.* Samples were matched for age, sex, and maternal education (as a proxy for socioeconomic status). Autistic sample: *M*_age_ = 12.3 years, *SD* = 3.08; non-autistic sample: *M*_age_ = 12.51 years, *SD* = 3.56. Paired-sample *t* test for age: *t* = 0.34, *p* = .733. χ^2^ test for sex (0 = male, 1 = female), maternal education (0 = no postsecondary education, 1 = postsecondary education), participants’ current education (0 = no special education, 1 = special education), intelligence level (0 = average, 1 = below/above average), comorbid condition (0 = no, 1 = yes), ethnicity (0 = German-speaking country, 1 = other), and native language (0 = monolingual, 1 = not monolingual). AD(H)D = attention deficit/hyperactivity disorder or attention deficit disorder.

**Table 3:** Reliabilities, Means, Standard Deviations, and *t* tests of the Cognitive Functions From the Intelligence and Development Scales–2 for Autistic and Non-Autistic Children and Adolescents.

Variable	Autistic Sample *N* = 43				Non-Autistic Sample *n* = 43				*t*	*df*	*p*	*p*_H_	*d*
	Rel	*M*	*SD*	Range	Rel	*M*	*SD*	Range					
Profile IQ ^b^	.99	90.16	19.98	55–131	.99	97.68	13.82	61–121	1.96	77	.027	.406	0.44
Full-Scale IQ ^b^	.99	91.58	20.77	55–129	.98	97.63	13.60	63–120	1.58	81	.059	.627	0.35
Screening IQ^b^	.98	93.54	19.35	55–125	.98	100.58	16.45	61–134	1.80	82	.038	.490	0.39
Visual Processing ^b^	.99	97.03	21.50	55–129	.97	102.67	12.50	80–129	1.46	80	.148	.809	0.32
Processing Speed ^b^	.98	95.58	20.47	55–143	.98	100.19	15.57	56–126	1.15	80	.126	.758	0.25
Auditory Short-Term Memory ^b^	.97	90.77	16.95	55–139	.96	97.76	12.54	64–121	2.12	79	.019	.352	0.47
Visuospatial Short-Term Memory ^b^	.97	88.92	15.12	55–118	.94	96.79	10.89	77–118	2.70	79	.004	.161	0.60
Abstract Reasoning ^b^	.98	95.10	20.67	55–141	.97	97.55	14.93	63–122	0.62	80	.539	.846	0.14
Verbal Reasoning ^b^	.99	94.32	19.29	58–126	.97	99.98	15.29	61–131	1.48	81	.071	.674	0.33
Long-Term Memory ^b^	.97	88.08	15.82	55–113	.97	93.64	16.06	58–137	1.57	79	.060	.627	0.35
Shape Design ^a^	.95	9.62	4.24	1–16	.89	10.65	2.55	7–16	1.36	83	.176	.846	0.30
Washer Design ^a^	.94	9.54	3.83	1–17	.92	10.57	2.78	4–19	1.41	81	.162	.811	0.31
Parrots ^a^	.92	9.15	4.28	1–19	.91	9.74	2.94	1–17	0.75	82	.228	.846	0.16
Boxes ^a^	.93	9.28	3.29	1–16	.90	10.40	3.36	2–17	1.54	80	.064	.642	0.34
Digit and Letter Span ^a^	.90	9.02	3.67	1–18	.82	9.95	2.17	5–14	1.42	83	.079	.693	0.31
Mixed Digit and Letter Span ^a^	.86	8.49	3.19	1–18	.84	10.21	2.99	1–17	2.51	79	.007	.231	0.56
Shape Memory ^a^	.88	8.41	2.99	1–14	.78	9.28	2.36	5–16	1.47	82	.072	.674	0.32
Rotated Shape Memory ^a^	.90	8.36	3.06	2–17	.82	10.12	2.63	6–18	2.78	79	.003	.132	0.62
Matrices: Completion ^a^	.93	9.32	3.63	3–18	.90	10.70	3.07	4–17	1.89	82	.063	.628	0.41
Matrices: Odd One Out ^a^	.93	9.72	3.82	2–18	.86	9.14	2.93	2–15	−0.78	80	.440	.846	0.17
Naming Categories ^a^	.95	9.38	3.92	1–16	.92	10.33	3.49	2–18	1.17	83	.122	.755	0.25
Naming Opposites ^a^	.92	9.41	3.49	1–16	.87	10.50	2.90	3–19	1.54	81	.063	.633	0.34
Story Recall ^a^	.93	8.40	3.56	1–14	.88	9.42	3.17	1–16	1.39	82	.084	.693	0.30
Picture Recall ^a^	.85	8.05	2.73	3–14	.88	8.90	3.32	3–18	1.27	80	.104	.726	0.28
Executive functions composite score ^b^	.97	8.84	2.20	4–13	.96	9.95	1.98	6–15	2.27	71	.013	.317	0.53
Listing Words ^c^	.75	7.89	3.13	1–14	.75	9.55	2.93	4–17	2.38	73	.010	.292	0.55
Divided Attention ^b^	.92	8.76	2.93	4–15	.90	10.11	2.52	5–17	2.13	71	.019	.352	0.50
Animal Colors ^c^	.72	8.09	3.55	1–14	.72	9.47	3.44	3–19	1.70	72	.047	.547	0.40
Drawing Routes ^b^	.96	9.91	2.60	5–15	.94	10.41	2.41	5–15	0.88	74	.192	.846	0.20

*Note.* Samples were matched for age, sex, and maternal education (as a proxy for socioeconomic status). *p*_H_ indicates *p* values adjusted with Hommel’s (1988) correction. Please note that after this correction, none of the comparisons were significant. Rel indicate reliabilities. The following reliabilities are reported: ^a^ Cronbach’s alpha, ^b^ reliability calculated according to a formula by Lienert and Raatz (1998), or ^c^ retest reliability.

**Table 4:** Reliabilities, Means, Standard Deviations, and *t* tests of the Developmental Functions From the Intelligence and Development Scales–2 for Autistic and Non-Autistic Children and Adolescents.

Variable	Autistic Sample *n* = 43				Non-Autistic Sample *n* = 43				*t*	*df*	*p*	*p*_H_	*d*
	Rel	*M*	*SD*	Range	Rel	*M*	*SD*	Range					
Psychomotor skills composite score ^b^	.98	8.49	2.22	4–12	.95	10.43	1.57	7–15	4.60	81	<.001	**<.001**	**1.01**
Gross Motor Skills ^a^	.72	5.29	3.57	1–11	.77	11.35	3.08	5–15	5.30	32	<.001	**<.001**	**1.82**
Fine Motor Skills ^b^	.96	8.65	3.03	2–14	.96	10.59	2.39	4–16	3.20	79	<.001	**.046**	**0.71**
Visuomotor Skills ^b^	.95	8.79	1.97	4–13	.87	10.01	1.73	7–13	3.01	81	.002	.077	0.66
Social–emotional skills composite score ^b^	.96	8.32	2.86	1–13	.95	9.82	2.15	5–13	2.71	82	.004	.154	0.59
Identifying Emotions ^c^	.85	7.71	4.27	1–12	.85	10.24	2.68	4–12	2.07	32	.023	.388	0.71
Regulating Emotions ^c^	.78	8.40	3.39	1–13	.78	10.00	2.75	4–15	2.37	82	.010	.292	0.52
Socially Competent Behavior ^c^	.71	8.32	3.17	1–15	.71	9.81	2.70	5–15	2.29	80	.012	.311	0.51
Basic skills composite score ^b^	.99	9.77	2.53	2–14	.99	9.92	2.15	5–13	0.29	74	.772	.846	0.07
Logical–Mathematical Reasoning ^a^	.98	9.07	4.21	1–17	.96	10.24	3.07	3–16	1.44	81	.153	.809	0.32
Language Skills ^b^	.98	7.12	2.65	3–14	.98	10.50	1.86	6–13	4.11	28	<.001	**.008**	**1.51**
Phoneme Analysis ^a^	.92	6.07	3.00	1–13	.97	10.24	3.13	3–15	3.75	29	<.001	**.019**	**1.35**
Phoneme–Grapheme Correspondence ^a^	.86	9.07	4.20	1–15	.96	10.00	2.18	5–13	0.79	29	.217	.846	0.29
Language Expressive ^a^	.81	7.08	2.96	1–14	.89	10.41	2.55	5–15	3.31	28	.001	.058	1.22
Language Receptive ^a^	.85	6.71	3.34	2–14	.81	11.35	2.37	7–16	4.52	29	<.001	**.003**	**1.63**
Reading ^b^	.98	8.76	3.33	1–15	.95	9.65	2.40	5–14	1.35	74	.182	.846	0.31
Reading Words ^c^	.79	9.34	3.41	2–16	.79	9.47	2.56	5–14	0.20	76	.846	.846	0.04
Reading Pseudo Words ^c^	.67	8.89	2.89	2–14	.67	9.82	2.48	4–14	1.52	75	.132	.792	0.35
Text Comprehension ^a^	.69	9.40	5.02	1–16	.69	10.37	2.79	4–16	1.00	68	.160	.809	0.24
Spelling ^a^	.88	8.89	3.19	3–15	.88	9.79	2.66	4–15	1.26	65	.212	.846	0.31
Motivation and attitude composite score ^b^	.96	10.56	3.24	6–17	.96	10.65	2.78	6–19	0.11	46	.458	.846	0.03
Conscientiousness ^a^	.82	10.21	3.27	6–18	.79	10.26	2.85	6–19	0.06	45	.477	.846	0.02
Achievement Motivation ^a^	.87	11.12	3.96	4–19	.86	11.04	3.11	6–19	−0.07	47	.528	.846	0.02
Participation during testing, intelligence ^a^	.93	8.19	3.15	1–16	.93	10.17	3.57	1–16	2.68	81	.004	.169	0.59
Participation during testing, executive functions ^a^	.89	8.76	2.75	1–16	.91	10.17	2.89	4–16	2.13	71	.018	.351	0.50
Participation during testing, developmental functions ^a^	.95	8.33	3.31	1–16	.92	10.66	3.02	5–16	3.30	79	<.001	**.035**	**0.73**

*Note.* Samples were matched for age, sex, and maternal education (as a proxy for socioeconomic status). *p*_H_ indicates *p* values adjusted with Hommel’s (1988) correction. Significant results after accounting for multiple testing (Hommel correction) are presented in bold. Rel indicate reliabilities. The following reliabilities are reported: ^a^ Cronbach’s alpha, ^b^ reliability calculated according to a formula by Lienert and Raatz (1998), or ^c^ retest reliability.

## Data Availability

The data presented in this study are available on request from the corresponding author. The data are not publicly available due to privacy issues and property rights.

## Notes

1 According to current models of intelligence (Schneider and McGrew 2018) and executive functions (Miyake et al. 2000), working memory can be understood as a component of intelligence or executive functions. Because working memory is included in the intelligence domain in the IDS-2, we subsumed working memory under the realm of intelligence.

2 Although the sample size met the robustness criteria for using independent-samples *t* tests (Eid et al. 2017), we also examined the variables regarding normal distribution and variance homogeneity. Analyses using the Shapiro–Wilk test showed that 12 of the 55 dependent variables may not fulfill the normality assumption. Therefore, we additionally calculated Mann–Whitney *U* tests for these variables. The results remained largely the same with two exceptions: First, the mean difference in the subtest Identifying Emotions was no longer significant before controlling for multiple testing. Second, the mean difference in the composite score of language skills was no longer significant after controlling for multiple testing. Furthermore, we found that the Levene’s test was significant for fewer than 10 of the dependent variables, indicating unequal variances. Thus, Welch’s *t* tests were additionally performed. The results were identical to those obtained from the independent-samples *t* tests.

3 To control for effects of intelligence, we repeated the independent-samples *t* tests for the developmental functions with a non-autistic control sample matched by age, sex, and intelligence (Full-Scale IQ). The pattern of results remained largely the same, showing lower group mean values for the autistic participants than for the control sample in the domains psychomotor skills, social–emotional skills, language skills, and participation during testing (see Table S2 in the Supplemental Material for full results). These differences hold when correcting for multiple testing in the domain of psychomotor skills. These post hoc analyses underscore the robustness of our findings.

## Supplementary Materials

The following supporting information can be downloaded at: https://www.mdpi.com/article/10.3390/jintelligence10040112/s1, Table S1: Description of the Composites, Group Factors, and Subtests of the Intelligence and Development Scales–2, Table S2: Means, Standard Deviations, and *t* tests of the Developmental Functions From the Intelligence and Development Scales–2 for Autistic and Non-Autistic Children and Adolescents Matched by Age, Sex, and Intelligence, Table S3: Means, Standard Deviations, and *t* tests of the Cognitive Functions From the Intelligence and Development Scales–2 for Autistic and Non-Autistic Children (Aged 5–10 Years), Table S4: Means, Standard Deviations, and *t* tests of the Developmental Functions From the Intelligence and Development Scales–2 for Autistic and Non-Autistic Children (Aged 5–10 Years), Table S5: Means, Standard Deviations, and *t* tests of the Cognitive Functions From the Intelligence and Development Scales–2 for Autistic and Non-Autistic Adolescents (Aged 11–20 Years), Table S6: Means, Standard Deviations, and *t* tests of the Developmental Functions From the Intelligence and Development Scales–2 for Autistic and Non-Autistic Adolescents (Aged 11–20 Years).

## References

[B1-jintelligence-10-00112] Akshoomoff Natacha (2006). Use of the Mullen Scales of Early Learning for the Assessment of Young Children with Autism Spectrum Disorders. Child Neuropsychology.

[B2-jintelligence-10-00112] American Psychiatric Association (2013). Diagnostic and Statistical Manual of Mental Disorders.

[B3-jintelligence-10-00112] Bhat Anjana N., Landa Rebecca J., Galloway James C. (Cole) (2011). Current Perspectives on Motor Functioning in Infants, Children, and Adults with Autism Spectrum Disorders. Physical Therapy.

[B4-jintelligence-10-00112] Boucher Jill (2012). Research Review: Structural Language in Autistic Spectrum Disorder—Characteristics and Causes. Journal of Child Psychology and Psychiatry.

[B5-jintelligence-10-00112] Bowen Sonya E. (2014). Autism Spectrum Disorders (ASD): State of the States of Services and Supports for People with ASD.

[B6-jintelligence-10-00112] Brignell Amanda, Morgan Angela T., Woolfenden Susan, Klopper Felicity, May Tamara, Sarkozy Vanessa, Williams Katrina (2018). A Systematic Review and Meta-Analysis of the Prognosis of Language Outcomes for Individuals with Autism Spectrum Disorder. Autism & Developmental Language Impairments.

[B7-jintelligence-10-00112] Brown Heather M., Oram-Cardy Janis, Johnson Andrew (2013). A Meta-Analysis of the Reading Comprehension Skills of Individuals on the Autism Spectrum. Journal of Autism and Developmental Disorders.

[B8-jintelligence-10-00112] Cai Ru Ying, Richdale Amanda L., Uljarević Mirko, Dissanayake Cheryl, Samson Andrea C. (2018). Emotion Regulation in Autism Spectrum Disorder: Where We Are and Where We Need to Go. Autism Research.

[B9-jintelligence-10-00112] Carnine Douglas W., Silbert Jerry, Kame’enui Edward J., Tarver Sara G. (2010). Direct Instruction Reading.

[B10-jintelligence-10-00112] Charman Tony, Pickles Andrew, Simonoff Emily, Chandler Susie, Loucas Tom, Baird Gillian (2011). IQ in Children with Autism Spectrum Disorders: Data from the Special Needs and Autism Project (SNAP). Psychological Medicine.

[B11-jintelligence-10-00112] Chiang Hsu-Min, Lin Yueh-Hsien (2007). Mathematical Ability of Students with Asperger Syndrome and High-Functioning Autism: A Review of Literature. Autism.

[B12-jintelligence-10-00112] Cibralic Sara, Kohlhoff Jane, Wallace Nancy, McMahon Catherine, Eapen Valsamma (2019). A Systematic Review of Emotion Regulation in Children with Autism Spectrum Disorder. Research in Autism Spectrum Disorders.

[B13-jintelligence-10-00112] Cohen Jacob (1988). Statistical Power Analysis for the Behavioral Sciences.

[B14-jintelligence-10-00112] Coll Sarah-Maude, Foster Nicholas E. V., Meilleur Alexa, Brambati Simona M., Hyde Krista L. (2020). Sensorimotor Skills in Autism Spectrum Disorder: A Meta-Analysis. Research in Autism Spectrum Disorders.

[B15-jintelligence-10-00112] Colombo-Dougovito Andrew M., Block Martin E. (2019). Fundamental Motor Skill Interventions for Children and Adolescents on the Autism Spectrum: A Literature Review. Review Journal of Autism and Developmental Disorders.

[B16-jintelligence-10-00112] Coolican Jamesie, Bryson Susan E., Zwaigenbaum Lonnie (2008). Brief Report: Data on the Stanford–Binet Intelligence Scales (5th Ed.) in Children with Autism Spectrum Disorder. Journal of Autism and Developmental Disorders.

[B17-jintelligence-10-00112] de Vries Marieke, Geurts Hilde M. (2012). Cognitive Flexibility in ASD; Task Switching with Emotional Faces. Journal of Autism and Developmental Disorders.

[B18-jintelligence-10-00112] Demetriou Eleni A., Lampit Amit, Quintana Daniel S., Naismith Sharon L., Song Yun J.C., Pye Julia E., Hickie Ian, Guastella Adam J. (2018). Autism Spectrum Disorders: A Meta-Analysis of Executive Function. Molecular Psychiatry.

[B19-jintelligence-10-00112] Dyck Murray J., Piek Jan P., Hay David, Smith Leigh, Hallmayer Joachim (2006). Are Abilities Abnormally Interdependent in Children With Autism?. Journal of Clinical Child & Adolescent Psychology.

[B20-jintelligence-10-00112] Dynia Jaclyn M., Bean Allison, Justice Laura M., Kaderavek Joan N. (2019). Phonological Awareness Emergence in Preschool Children with Autism Spectrum Disorder. Autism & Developmental Language Impairments.

[B21-jintelligence-10-00112] Eid Michael, Gollwitzer Mario, Schmitt Manfred (2017). Statistik und Forschungsmethoden [Statistics and Research Methods].

[B22-jintelligence-10-00112] Ellis Weismer Susan, Lord Catherine, Esler Amy (2010). Early Language Patterns of Toddlers on the Autism Spectrum Compared to Toddlers with Developmental Delay. Journal of Autism and Developmental Disorders.

[B23-jintelligence-10-00112] Fatemi S. Hossein, Aldinger Kimberly A., Ashwood Paul, Bauman Margaret L., Blaha Charles D., Blatt Gene J., Chauhan Abha, Chauhan Ved, Dager Stephen R., Dickson Price E. (2012). Consensus Paper: Pathological Role of the Cerebellum in Autism. The Cerebellum.

[B24-jintelligence-10-00112] Faul Franz, Erdfelder Edgar, Lang Albert-Georg, Buchner Axel (2007). G*Power 3: A Flexible Statistical Power Analysis Program for the Social, Behavioral, and Biomedical Sciences. Behavior Research Methods.

[B25-jintelligence-10-00112] Finnegan Elizabeth, Accardo Amy L. (2018). Written Expression in Individuals with Autism Spectrum Disorder: A Meta-Analysis. Journal of Autism and Developmental Disorders.

[B26-jintelligence-10-00112] Georgiou Alexandra, Soulis Spyridon-Georgios, Rapti Danai (2018). Motivation in Mathematics of High Functioning Students With Autism Spectrum Disorder (ASD). Journal of Psychology Research.

[B27-jintelligence-10-00112] Grieder Silvia, Grob Alexander (2020). Exploratory Factor Analyses of the Intelligence and Development Scales–2: Implications for Theory and Practice. Assessment.

[B28-jintelligence-10-00112] Grob Alexander, Hagmann-von Arx Priska (2018a). Intelligence and Development Scales–2 (IDS-2). Intelligenz- und Entwicklungsskalen für Kinder und Jugendliche [Intelligence and Development Scales for Children and Adolescents].

[B29-jintelligence-10-00112] Grob Alexander, Hagmann-von Arx Priska (2018b). Intelligence and Development Scales–2 (IDS-2). Intelligenz- und Entwicklungsskalen für Kinder und Jugendliche. Manual zu Theorie, Interpretation und Gütekriterien [Intelligence and Development Scales for Children and Adolescents. Manual on Theory, Interpretation and Psychometric Criteria].

[B30-jintelligence-10-00112] Grob Alexander, Arx Priska Hagmann-von, Jaworowska Aleksandra, Matczak Anna, Fecenec Diana (2019). Intelligence and Development Scales-2. Inteligencji i Rozwoju Dla Dzieci i Młodzieży [Intelligence and Development Scales for Children and Adolescents].

[B31-jintelligence-10-00112] Grob Alexander, Arx Priska Hagmann-von, Barnett Anna, Stuart Nichola, Vanzan Serena (2021). Intelligence and Development Scales-2 (IDS-2). Intelligence and Development Scales for Children and Adolescents.

[B32-jintelligence-10-00112] Grob Alexander, Arx Priska Hagmann-von, Ferri Rosa, Rea Monica, Casagrande Maria (2022). Intelligence and Development Scales-2. Scale Di Intelligenza e Sviluppo per Bambini e Adolescenti [Intelligence and Development Scales for Children and Adolescents].

[B33-jintelligence-10-00112] Grob Alexander, Arx Priska Hagmann-von, Ruiter Selma A. J., Timmerman Marieke E., Visser Linda (2018). Intelligence and Development Scales–2 (IDS-2). Intelligentie- En Ontwikkelingsschalen Voor Kinderen En Jongeren. [Intelligence and Development Scales for Children and Adolescents].

[B34-jintelligence-10-00112] Grondhuis Sabrina N., Lecavalier Luc, Arnold L. Eugene, Handen Benjamin L., Scahill Lawrence, McDougle Christopher J., Aman Michael G. (2018). Differences in Verbal and Nonverbal IQ Test Scores in Children with Autism Spectrum Disorder. Research in Autism Spectrum Disorders.

[B35-jintelligence-10-00112] Hannant Penelope, Tavassoli Teresa, Cassidy Sarah (2016). The Role of Sensorimotor Difficulties in Autism Spectrum Conditions. Frontiers in Neurology.

[B36-jintelligence-10-00112] Happé Francesca, Frith Uta (2020). Annual Research Review: Looking Back to Look Forward—Changes in the Concept of Autism and Implications for Future Research. Journal of Child Psychology and Psychiatry.

[B37-jintelligence-10-00112] Harms Madeline B., Martin Alex, Wallace Gregory L. (2010). Facial Emotion Recognition in Autism Spectrum Disorders: A Review of Behavioral and Neuroimaging Studies. Neuropsychology Review.

[B38-jintelligence-10-00112] Hill Elisabeth L. (2004). Executive Dysfunction in Autism. Trends in Cognitive Sciences.

[B39-jintelligence-10-00112] Ho Daniel E., Imai Kosuke, King Gary, Stuart Elizabeth A. (2011). MatchIt: Nonparametric Preprocessing for Parametric Causal Inference. Journal of Statistical Software.

[B40-jintelligence-10-00112] Hommel Gerhard (1988). A Stagewise Rejective Multiple Test Procedure Based on a Modified Bonferroni Test. Biometrika.

[B41-jintelligence-10-00112] Howlin Patricia, Moss Philippa (2012). Adults with Autism Spectrum Disorders. The Canadian Journal of Psychiatry.

[B42-jintelligence-10-00112] Hudry Kristelle, Leadbitter Kathy, Temple Kathryn, Slonims Vicky, McConachie Helen, Aldred Catherine, Howlin Patricia, Charman Tony, Consortium the PACT (2010). Preschoolers with Autism Show Greater Impairment in Receptive Compared with Expressive Language Abilities. International Journal of Language & Communication Disorders.

[B43-jintelligence-10-00112] Idring Selma, Lundberg Michael, Sturm Harald, Dalman Christina, Gumpert Clara, Rai Dheeraj, Lee Brian K., Magnusson Cecilia (2015). Changes in Prevalence of Autism Spectrum Disorders in 2001–2011: Findings from the Stockholm Youth Cohort. Journal of Autism and Developmental Disorders.

[B44-jintelligence-10-00112] Jahromi Laudan B., Meek Shantel E., Ober-Reynolds Sharman (2012). Emotion Regulation in the Context of Frustration in Children with High Functioning Autism and Their Typical Peers. Journal of Child Psychology and Psychiatry.

[B45-jintelligence-10-00112] Jones Catherine R. G., Pickles Andrew, Falcaro Milena, Marsden Anita J. S., Happé Francesca, Scott Sophie K., Sauter Disa, Tregay Jenifer, Phillips Rebecca J., Baird Gillian (2011). A Multimodal Approach to Emotion Recognition Ability in Autism Spectrum Disorders. Journal of Child Psychology and Psychiatry.

[B46-jintelligence-10-00112] Keen Deb (2009). Engagement of Children with Autism in Learning. Australasian Journal of Special Education.

[B47-jintelligence-10-00112] Keen Deb, Webster Amanda, Ridley Greta (2016). How Well Are Children with Autism Spectrum Disorder Doing Academically at School? An Overview of the Literature. Autism.

[B48-jintelligence-10-00112] Kjellmer Liselotte, Fernell Elisabeth, Gillberg Christopher, Norrelgen Fritjof (2018). Speech and Language Profiles in 4- to 6-Year-Old Children with Early Diagnosis of Autism Spectrum Disorder without Intellectual Disability. Neuropsychiatric Disease and Treatment.

[B49-jintelligence-10-00112] Konstantareas M. Mary, Stewart Kelly (2006). Affect Regulation and Temperament in Children with Autism Spectrum Disorder. Journal of Autism and Developmental Disorders.

[B50-jintelligence-10-00112] Kurth Jennifer A., Mastergeorge Ann M. (2010). Academic and Cognitive Profiles of Students with Autism: Implications for Classroom Practice and Placement. International Journal of Special Education.

[B51-jintelligence-10-00112] Kwok Elaine Y. L., Brown Heather M., Smyth Rachael E., Cardy Janis Oram (2015). Meta-Analysis of Receptive and Expressive Language Skills in Autism Spectrum Disorder. Research in Autism Spectrum Disorders.

[B52-jintelligence-10-00112] Leung Florence Yik Nam, Sin Jacqueline, Dawson Caitlin, Ong Jia Hoong, Zhao Chen, Veić Anamarija, Liu Fang (2022). Emotion Recognition across Visual and Auditory Modalities in Autism Spectrum Disorder: A Systematic Review and Meta-Analysis. Developmental Review.

[B53-jintelligence-10-00112] Leyfer Ovsanna T., Folstein Susan E., Bacalman Susan, Davis Naomi O., Dinh Elena, Morgan Jubel, Tager-Flusberg Helen, Lainhart Janet E. (2006). Comorbid Psychiatric Disorders in Children with Autism: Interview Development and Rates of Disorders. Journal of Autism and Developmental Disorders.

[B54-jintelligence-10-00112] Libertus Klaus, Sheperd Kelly A., Ross Samuel W., Landa Rebecca J. (2014). Limited Fine Motor and Grasping Skills in 6-Month-Old Infants at High Risk for Autism. Child Development.

[B55-jintelligence-10-00112] Lienert Gustav A., Raatz Ulrich (1998). Testaufbau und Testanalyse [Test Design and Test Analysis].

[B56-jintelligence-10-00112] Liu Ting, Breslin Casey M. (2013). Fine and Gross Motor Performance of the MABC-2 by Children with Autism Spectrum Disorder and Typically Developing Children. Research in Autism Spectrum Disorders.

[B57-jintelligence-10-00112] Lodi-Smith Jennifer, Rodgers Jonathan D., Cunningham Sara A., Lopata Christopher, Thomeer Marcus L. (2019). Meta-Analysis of Big Five Personality Traits in Autism Spectrum Disorder. Autism.

[B58-jintelligence-10-00112] Luyster Rhiannon, Lopez Kristina, Lord Catherine (2007). Characterizing Communicative Development in Children Referred for Autism Spectrum Disorders Using the MacArthur-Bates Communicative Development Inventory (CDI). Journal of Child Language.

[B59-jintelligence-10-00112] MacDonald Megan, Lord Catherine, Ulrich Dale A. (2013a). The Relationship of Motor Skills and Adaptive Behavior Skills in Young Children with Autism Spectrum Disorders. Research in Autism Spectrum Disorders.

[B60-jintelligence-10-00112] MacDonald Megan, Lord Catherine, Ulrich Dale A. (2013b). The Relationship of Motor Skills and Social Communicative Skills in School-Aged Children with Autism Spectrum Disorder. Adapted Physical Activity Quarterly.

[B61-jintelligence-10-00112] Maenner Matthew J., Shaw Kelly A., Baio Jon, Washington Anita, Patrick Mary, DiRienzo Monica, Christensen Deborah L., Wiggins Lisa D., Pettygrove Sydney, Andrews Jennifer G. (2020). Prevalence of Autism Spectrum Disorder among Children Aged 8 Years—Autism and Developmental Disabilities Monitoring Network, 11 Sites, United States, 2016. MMWR Surveillance Summaries.

[B62-jintelligence-10-00112] Mandelbaum David E., Stevens Michael, Rosenberg Eric, Wiznitzer Max, Steinschneider Mitchell, Korey Saul R., Filipek Pauline, Rapin Isabelle, Korey Saul R. (2006). Sensorimotor Performance in School-Age Children with Autism, Developmental Language Disorder, or Low IQ. Developmental Medicine & Child Neurology.

[B63-jintelligence-10-00112] Manicolo Olivia, Brotzmann Mark, Arx Priska Hagmann-von, Grob Alexander, Weber Peter (2019). Gait in Children with Infantile/Atypical Autism: Age-Dependent Decrease in Gait Variability and Associations with Motor Skills. European Journal of Paediatric Neurology.

[B64-jintelligence-10-00112] Mayes Susan Dickerson, Calhoun Susan L. (2003a). Analysis of WISC-III, Stanford-Binet:IV, and Academic Achievement Test Scores in Children with Autism. Journal of Autism and Developmental Disorders.

[B65-jintelligence-10-00112] Mayes Susan Dickerson, Calhoun Susan L. (2003b). Ability Profiles in Children with Autism: Influence of Age and IQ. Autism.

[B66-jintelligence-10-00112] Mazefsky Carla A., Borue Xenia, Day Taylor N., Minshew Nancy J. (2014). Emotion Regulation Patterns in Adolescents with High-Functioning Autism Spectrum Disorder: Comparison to Typically Developing Adolescents and Association with Psychiatric Symptoms. Autism Research.

[B67-jintelligence-10-00112] Meyer Christine Sandra, Arx Priska Hagmann-von, Grob Alexander (2009). Die Intelligence and Development Scale Sozial-Emotionale Kompetenz (IDS-SEK): Psychometrische Eigenschaften eines Tests zur Erfassung sozial-emotionaler Fähigkeiten [The Intelligence and Development Scale Social-Emotional Competence (IDS-SEK): Psychometric properties of a test to assess social-emotional skills]. Diagnostica.

[B68-jintelligence-10-00112] Ming Xue, Brimacombe Michael, Wagner George C. (2007). Prevalence of Motor Impairment in Autism Spectrum Disorders. Brain and Development.

[B69-jintelligence-10-00112] Mitchell Shelley, Cardy Janis Oram, Zwaigenbaum Lonnie (2011). Differentiating Autism Spectrum Disorder from Other Developmental Delays In The First Two Years Of Life. Developmental Disabilities Research Reviews.

[B70-jintelligence-10-00112] Mitchell Shelley, Brian Jessica, Zwaigenbaum Lonnie, Roberts Wendy, Szatmari Peter, Smith Isabel, Bryson Susan (2006). Early Language and Communication Development of Infants Later Diagnosed with Autism Spectrum Disorder. Journal of Developmental & Behavioral Pediatrics.

[B71-jintelligence-10-00112] Miyake Akira, Friedman Naomi P. (2012). The Nature and Organization of Individual Differences in Executive Functions: Four General Conclusions. Current Directions in Psychological Science.

[B72-jintelligence-10-00112] Miyake Akira, Friedman Naomi P., Emerson Michael J., Witzki Alexander H., Howerter Amy, Wager Tor D. (2000). The Unity and Diversity of Executive Functions and Their Contributions to Complex ‘Frontal Lobe’ Tasks: A Latent Variable Analysis. Cognitive Psychology.

[B73-jintelligence-10-00112] Mostofsky Stewart H., Burgess Melanie P., Larson Jennifer C. Gidley (2007). Increased Motor Cortex White Matter Volume Predicts Motor Impairment in Autism. Brain.

[B74-jintelligence-10-00112] Mostofsky Stewart H., Powell Stephanie K., Simmonds Daniel J., Goldberg Melissa C., Caffo Brian, Pekar James J. (2009). Decreased Connectivity and Cerebellar Activity in Autism during Motor Task Performance. Brain.

[B75-jintelligence-10-00112] Muth Anne, Hönekopp Johannes, Falter Christine M. (2014). Visuo-Spatial Performance in Autism: A Meta-Analysis. Journal of Autism and Developmental Disorders.

[B76-jintelligence-10-00112] Nader Anne-Marie, Courchesne Valérie, Dawson Michelle, Soulières Isabelle (2016). Does WISC-IV Underestimate the Intelligence of Autistic Children?. Journal of Autism and Developmental Disorders.

[B77-jintelligence-10-00112] Nagy Emese, Prentice Louise, Wakeling Tess (2021). Atypical Facial Emotion Recognition in Children with Autism Spectrum Disorders: Exploratory Analysis on the Role of Task Demands. Perception.

[B78-jintelligence-10-00112] Nebel Mary Beth, Joel Suresh E., Muschelli John, Barber Anita D., Caffo Brian S., Pekar James J., Mostofsky Stewart H. (2014). Disruption of Functional Organization within the Primary Motor Cortex in Children with Autism. Human Brain Mapping.

[B79-jintelligence-10-00112] Oliveira Katherine Simone Caires, Fontes Déborah Ebert, Longo Egmar, Leite Hércules Ribeiro, Camargos Ana Cristina Resende (2021). Motor Skills Are Associated with Participation of Children with Autism Spectrum Disorder. Journal of Autism and Developmental Disorders.

[B80-jintelligence-10-00112] Oliveras-Rentas Rafael E., Kenworthy Lauren, Roberson Richard B., Martin Alex, Wallace Gregory L. (2012). WISC-IV Profile in High-Functioning Autism Spectrum Disorders: Impaired Processing Speed Is Associated with Increased Autism Communication Symptoms and Decreased Adaptive Communication Abilities. Journal of Autism and Developmental Disorders.

[B81-jintelligence-10-00112] Pennington Bruce F., Ozonoff Sally (1996). Executive Functions and Developmental Psychopathology. Journal of Child Psychology and Psychiatry.

[B82-jintelligence-10-00112] Petermann Franz (2008). Movement Assessment Battery for Children.

[B83-jintelligence-10-00112] Provost Beth, Lopez Brian R., Heimerl Sandra (2007). A Comparison of Motor Delays in Young Children: Autism Spectrum Disorder, Developmental Delay, and Developmental Concerns. Journal of Autism and Developmental Disorders.

[B84-jintelligence-10-00112] R Core Team (2021). R: A Language and Environment for Statistical Computing (Version 4.0.3) [Computer Software].

[B85-jintelligence-10-00112] Rosset Delphine B., Rondan Cécilie, Fonseca David Da, Santos Andreia, Assouline Brigitte, Deruelle Christine (2008). Typical Emotion Processing for Cartoon but Not for Real Faces in Children with Autistic Spectrum Disorders. Journal of Autism and Developmental Disorders.

[B86-jintelligence-10-00112] Russell Ginny, Mandy William, Elliott Daisy, White Rhianna, Pittwood Tom, Ford Tamsin (2019). Selection Bias on Intellectual Ability in Autism Research: A Cross-Sectional Review and Meta-Analysis. Molecular Autism.

[B87-jintelligence-10-00112] Salazar Fernando, Baird Gillian, Chandler Susie, Tseng Evelin, O’sullivan Tony, Howlin Patricia, Pickles Andrew, Simonoff Emily (2015). Co-Occurring Psychiatric Disorders in Preschool and Elementary School-Aged Children with Autism Spectrum Disorder. Journal of Autism and Developmental Disorders.

[B88-jintelligence-10-00112] Salomone Erica, Bulgarelli Daniela, Thommen Evelyne, Rossini Emanuelle, Molina Paola (2019). Role of Age and IQ in Emotion Understanding in Autism Spectrum Disorder: Implications for Educational Interventions. European Journal of Special Needs Education.

[B89-jintelligence-10-00112] Schmidt-Atzert Lothar, Amelang Manfred (2012). Psychologische Diagnostik [Psychological Assessment].

[B90-jintelligence-10-00112] Schneider W. Joel, McGrew Kevin S., Flanagan Dawn P., McDonough Erin M. (2018). The Cattell–Horn–Carroll Theory of Cognitive Abilities. Contemporary Intellectual Assessment: Theories, Tests, and Issues.

[B91-jintelligence-10-00112] Shanok Nathaniel A., Jones Nancy Aaron, Lucas Nikola N. (2019). The Nature of Facial Emotion Recognition Impairments in Children on the Autism Spectrum. Child Psychiatry & Human Development.

[B92-jintelligence-10-00112] Siaperas Panagiotis, Ring Howard A., McAllister Catherine J., Henderson Sheila, Barnett Anna, Watson Peter, Holland Anthony J. (2012). Atypical Movement Performance and Sensory Integration in Asperger’s Syndrome. Journal of Autism and Developmental Disorders.

[B93-jintelligence-10-00112] Thomas Pauline, Zahorodny Walter, Peng Bo, Kim Soyeon, Jani Nisha, Halperin William, Brimacombe Michael (2012). The Association of Autism Diagnosis with Socioeconomic Status. Autism.

[B94-jintelligence-10-00112] Titeca Daisy, Roeyers Herbert, Desoete Annemie (2017). Early Numerical Competencies in 4- and 5-Year-Old Children with Autism Spectrum. Focus on Autism and Other Developmental Disabilities.

[B95-jintelligence-10-00112] Trevisan Dominic A., Birmingham Elina (2016). Are Emotion Recognition Abilities Related to Everyday Social Functioning in ASD? A Meta-Analysis. Research in Autism Spectrum Disorders.

[B96-jintelligence-10-00112] Troyb Eva, Orinstein Alyssa, Tyson Katherine, Helt Molly, Eigsti Inge-Marie, Stevens Michael, Fein Deborah (2014). Academic Abilities in Children and Adolescents with a History of Autism Spectrum Disorders Who Have Achieved Optimal Outcomes. Autism.

[B97-jintelligence-10-00112] Van Meter Karla C., Christiansen Lasse E., Delwiche Lora D., Azari Rahman, Carpenter Tim E., Hertz-Picciotto Irva (2010). Geographic Distribution of Autism in California: A Retrospective Birth Cohort Analysis. Autism Research.

[B98-jintelligence-10-00112] Volden Joanne, Smith Isabel M., Szatmari Peter, Bryson Susan, Fombonne Eric, Mirenda Pat, Roberts Wendy, Vaillancourt Tracy, Waddell Charlotte, Zwaigenbaum Lonnie (2011). Using the Preschool Language Scale, Fourth Edition to Characterize Language in Preschoolers with Autism Spectrum Disorders. American Journal of Speech-Language Pathology.

[B99-jintelligence-10-00112] White Susan Williams, Scahill Lawrence, Klin Ami, Koenig Kathleen, Volkmar Fred R. (2007). Educational Placements and Service Use Patterns of Individuals with Autism Spectrum Disorders. Journal of Autism and Developmental Disorders.

[B100-jintelligence-10-00112] World Health Organization (2016). International Statistical Classification of Diseases and Related Health Problems (10th Rev.).

[B101-jintelligence-10-00112] World Health Organization (2018). International Classification of Diseases for Mortality and Morbidity Statistics (11th Rev.).

[B102-jintelligence-10-00112] Yerys Benjamin E., Wallace Gregory L., Sokoloff Jennifer L., Shook Devon A., James Joette D., Kenworthy Lauren (2009). Attention Deficit/Hyperactivity Disorder Symptoms Moderate Cognition and Behavior in Children with Autism Spectrum Disorders. Autism Research.

[B103-jintelligence-10-00112] Yeung Michael K. (2022). A Systematic Review and Meta-Analysis of Facial Emotion Recognition in Autism Spectrum Disorder: The Specificity of Deficits and the Role of Task Characteristics. Neuroscience & Biobehavioral Reviews.

[B104-jintelligence-10-00112] Zajic Matthew C., Solari Emily J., Grimm Ryan P., McIntyre Nancy S., Mundy Peter C. (2020). Relationships between Reading Profiles and Narrative Writing Abilities in School-Age Children with Autism Spectrum Disorder. Reading and Writing.

