# Supplementary material for: Cognitive and Developmental Functions in Autistic and Non-Autistic Children and Adolescents: Evidence from the Intelligence and Development Scales–2
Source: J Intell. 2022 Nov 21;10(4):112. doi: 10.3390/jintelligence10040112 (PMC9680381; doi:10.3390/jintelligence10040112)
Supplement: Supplementary file 1 [file jintelligence-10-00112-s001.zip › jintelligence-1989732-supplementary.pdf]

Supplementary Material to "Cognitive and Developmental Functions in Autistic and Non-Autistic Children and Adolescents: Evidence from the Intelligence and Development Scales–2"

Table S1

Description of the Composites, Group Factors, and Subtests of the Intelligence and Development Scales–2

Composite	Group factor	Subtest/Task	No. of items/subtests	Description	Measurement of
Profile IQ (PrIQ)			14	Including the subtests SD, WD, PSP, PSB, DLS, MDLS, SM, RSM, MC, MOO, NC, NO, SR, PR	
Full-Scale IQ (FSIQ)			7	Including one subtest from every group factor (i.e., SD, PSP, DLS, SM, MC, NC, SR)	
Screening IQ (ScrIQ)			2	Including one subtest from the group factor AR (i.e., MC) and one subtest from the group factor VR (i.e., NC)	
	Visual Processing (VP)	Shape Design (SD)	20	Reproduce geometric figures with the help of rectangles and triangles	Visual processing
		Washer Design (WD)	2–4[a]	Reproduce counter patterns according to a template	Visual processing
	Processing Speed (PS)	Parrots (PSP)	56–180[a]	Cross out parrots with two orange features that look to the left from rows of different parrots	Processing speed
		Boxes (PSB)	104–180[a]	Cross out groups of three or four boxes from rows of different groups of boxes	Processing speed
	Auditory Short-Term Memory (ASTM)	Digit and Letter Span (DLS)	40	Repeat number and letter sequences forward and backward	Forward: Short-term memory<br>Backward: Working memory
		Mixed Digit and Letter Span (MDLS)	36	Repeat mixed number and letter sequences forward and backward	Forward: Short-term memory<br>Backward: Working memory
	Visuospatial Short-Term Memory (VSTM)	Shape Memory (SM)	23	Remember figures and recognize them from a selection of figures and positions	Short-term memory
		Rotated Shape Memory (RSM)	23	Remember figures and recognize them from a selection of rotated figures and positions	Working memory
	Abstract Reasoning (AR)	Matrices: Completion (MC)	35	Understand how a figure changes and transfer these changes to a continuing figure	Fluid reasoning
		Matrices: Odd One Out (MOO)	31	Select from presented pictures the one that does not fit with the others	Fluid reasoning
	Verbal Reasoning (VR)	Naming Categories (NC)	34	Name categories for a group of pictures or words	Comprehension-knowledge
		Naming Opposites (NO)	34	Name opposites of presented words	Comprehension-knowledge
	Long-Term Memory (LTM)	Story Recall (SR)	19–32[a]	Listen to a semantically meaningful story and recall it after at least 20 min	Long-term memory
		Picture Recall (PR)	11–21[a]	Look at a picture and recall key features and details after at least 20 min	Long-term memory
Executive functions composite score (EFC)			4	Including the subtests LW, DA, AC, DR	
		Listing Words (LW)	2-4[a]	List words based on categories or starting letters	Word fluency
		Divided Attention (DA)	50-100[a]	Cross out parrots with two orange features that look to the left from different parrots and list animals at the same time	Cognitive flexibility
		Animal Colors (AC)	3	Say colors of animals as fast as possible	Inhibition
		Drawing Routes (DR)	14	Travel given routes as fast as possible once	Planning
Psychomotor skills composite score (PSC)			3	Including the subtests GM, FM, VM	

Composite	Group factor	Subtest/Task	No. of items/subtests	Description	Measurement of
		Gross Motor Skills (GM)	3	Balance on a rope, catch and throw a ball, and jump sideways over a rope	Gross motor skills
		Fine Motor Skills (FM)	6	Quickly screw nuts on and off bolts of different sizes and quickly thread beads of different sizes	Fine motor skills
		Visuomotor Skills (VM)	12	Move exactly between lines, draw figures, and reflect figures	Visuomotor skills
Social-emotional skills composite score (SESC)			3	Including the subtests IE, RE, SC	
		Identifying Emotions (IE)	10	Recognize and name emotions of children in photos	Emotion recognition
		Regulating Emotions (RE)	6-9[a]	Specify regulation strategies for the emotions of anger, fear, and grief	Emotion regulation
		Socially Competent Behavior (SC)	6-9[a]	Name socially competent behavior according to a presented social situation	Socially competent behavior
Basic skills composite score (BSC)			4	Including the subtests MR, LS, RD, SP	
		Logical-Mathematical Reasoning (MR)	64	Solve logical-mathematical reasoning tasks	Mathematical skills
		Language Skills (LS)	4	Including the tasks PA, PGC, LE, LR	Language skills
		Phoneme Analysis (PA)	56	Clap syllables, recognize rhymes, isolate on and off sounds, sound out words	Phonological awareness
		Phoneme–Grapheme Correspondence (PGC)	42	Match phonemes and graphemes, recognize short and long vowels	Knowledge of letter–sound correspondence
		Language Expressive (LE)	12	Form sentences from several words	Language production
		Language Receptive (LR)	15	Carry out instructions	Language comprehension
		Reading (RD)	3	Including RW, RP, TC	Reading
		Reading Words (RW)	60	Read real words	Word recognition
		Reading Pseudo Words (RP)	60	Read pseudo words	Synthetic reading
		Text Comprehension (TC)	14-19[a]	Read and understand texts	Reading comprehension
		Spelling (SP)	40-60[a]	Word dictation	Spelling skills
Motivation and attitude composite score (MAC)			2	Including the subtests CS, AM	
		Conscientiousness (CS)	18	Rate statements regarding conscientiousness	Conscientiousness
		Achievement Motivation (AM)	18	Rate statements regarding achievement motivation	Achievement motivation
		Participation during testing, intelligence (PDTIQ)	12	The test administrator evaluates the participant's cooperation during testing of the intelligence domain	Achievement motivation
		Participation during testing, executive functions (PDTEF)	12	The test administrator evaluates the participant's cooperation during testing of the executive functions domain	Achievement motivation
		Participation during testing, developmental functions (PDTDF)	12	The test administrator evaluates the participant's cooperation during testing of the developmental functions domain	Achievement motivation

Note. Gross Motor Skills, Identifying Emotions, and Language Skills only for ages 5 to 10 years; Reading and Spelling only for ages 7 to 20 years; Conscientiousness and Achievement Motivation only for ages 11 to 20 years.

[a] Depending on age and skill.

Table S2

Means, Standard Deviations, and t *tests of the Developmental Functions From the Intelligence and Development Scales–2 for Autistic and Non-Autistic Children and Adolescents Matched by Age, Sex, and Intelligence*

Variable	Autistic sample $n = 43$			Non-autistic sample $n = 43$			t	df	p	p_H	d
	M	SD	Range	M	SD	Range					
Psychomotor skills composite score	8.49	2.22	4–12	10.15	2.03	6–15	3.58	82	< .001	**.007**	**0.78**
Gross Motor Skills	5.29	3.57	1–11	10.31	3.09	4–17	4.31	31	< .001	**.002**	**1.50**
Fine Motor Skills	8.65	3.03	2–14	10.19	2.81	2–17	2.41	81	.009	.166	0.53
Visuomotor Skills	8.79	1.97	4–13	10.16	1.81	7–15	3.29	80	< .001	**.018**	**0.73**
Social-emotional skills composite score	8.32	2.86	1–13	9.70	2.19	4–15	2.50	83	.007	.135	0.54
Identifying Emotions	7.71	4.27	1–12	9.41	3.18	1–12	1.32	32	.098	.884	0.45
Regulating Emotions	8.40	3.39	1–13	9.35	3.01	1–15	1.36	83	.089	.884	0.30
Socially Competent Behavior	8.32	3.17	1–15	9.98	2.92	6–18	2.45	80	.008	.155	0.54
Basic skills composite score	9.77	2.53	2–14	9.01	3.09	1–13	-1.15	75	.254	.999	0.26
Logical-Mathematical Reasoning	9.07	4.21	1–17	9.63	3.86	1–15	0.63	82	.531	.999	0.14
Language Skills	7.12	2.65	3–14	9.01	3.44	2–14	1.65	28	.055	.662	0.61
Phoneme Analysis	6.07	3.00	1–13	9.18	4.05	1–16	2.38	29	.012	.218	0.86
Phoneme–Grapheme Correspondence	9.07	4.20	1–15	9.41	4.05	1–17	0.23	29	.410	.999	0.08
Language Expressive	7.08	2.96	1–14	9.47	4.85	1–16	1.57	28	.064	.762	0.58
Language Receptive	6.71	3.34	2–14	8.00	3.76	1–14	1.00	29	.164	.999	0.36
Reading	8.76	3.33	1–15	8.76	2.95	1–14	0.00	71	.999	.999	0.00
Reading Words	9.34	3.41	2–16	8.46	3.00	1–14	-1.19	73	.238	.999	0.27
Reading Pseudo Words	8.89	2.89	2–14	8.89	3.06	1–16	0.00	72	.999	.999	0.00
Text Comprehension	9.40	5.02	1–16	10.21	3.62	2–16	0.72	62	.236	.999	0.18
Spelling	8.89	3.19	3–15	8.29	3.76	1–14	-0.67	60	.507	.999	0.17
Motivation and attitude composite score	10.56	3.24	6–17	10.20	2.57	5–19	-0.45	51	.674	.999	0.13
Conscientiousness	10.21	3.27	6–18	10.25	2.49	6–19	0.05	50	.479	.999	0.01

Variable	Autistic sample $n = 43$			Non-autistic sample $n = 43$			t	df	p	p_H	d
	M	*SD*	Range	*M*	*SD*	Range					
Achievement Motivation	11.12	3.96	4–19	10.14	3.25	1–19	-0.99	52	.836	.999	0.27
Participation during testing, intelligence	8.19	3.15	1–16	9.74	3.05	2–16	2.31	83	.012	.210	0.50
Participation during testing, executive functions	8.76	2.75	1–16	9.53	2.83	2–15	1.21	75	.116	.955	0.28
Participation during testing, developmental functions	8.33	3.31	1–16	9.85	3.08	3–17	2.14	80	.018	.315	0.47

Note. Samples were matched for age, sex, and intelligence (Full-Scale IQ). p_H indicates p values adjusted with Hommel's (1988) correction. Significant results after accounting for multiple testing (Hommel correction) are presented in bold.

Table S3

Means, Standard Deviations, and t *tests of the Cognitive Functions From the Intelligence and Development Scales–2 for Autistic and Non-Autistic Children (Aged 5-10 Years)*

Variable	Autistic children $n = 17$			Non-autistic children $n = 17$			*t*	*df*	*p*	p_H	*d*
	M	*SD*	Range	*M*	*SD*	Range					
Profile IQ[b]	77.69	20.08	55–114	100.93	13.49	74–118	3.64	26	< .001	**.017**	**1.38**
Full-Scale IQ[b]	79.53	20.55	55–119	99.65	14.99	74–120	3.19	30	.002	**.033**	**1.13**
Screening IQ[b]	83.12	18.47	55–122	101.59	16.54	70–134	3.03	31	.002	**.037**	**1.06**
Visual Processing[b]	84.21	27.23	55–129	102.62	11.65	80–117	2.46	28	.020	.115	0.90
Processing Speed[b]	83.53	20.75	55–131	101.62	19.10	56–125	2.53	29	.009	.070	0.91
Auditory Short-Term Memory[b]	81.15	12.53	61–97	96.06	15.13	64–120	2.84	27	.004	**.047**	**1.06**
Visuospatial Short-Term Memory[b]	81.64	15.99	55–105	99.56	11.42	77–117	3.57	28	< .001	**.019**	**1.31**
Abstract Reasoning[b]	84.80	21.49	55–141	98.75	13.78	73–119	2.17	29	.039	.155	0.78
Verbal Reasoning[b]	85.00	17.74	61–123	102.75	14.01	78–131	3.10	29	.002	**.036**	**1.12**
Long-Term Memory[b]	85.50	16.26	55–107	97.62	13.84	67–125	2.21	28	.018	.107	0.81
Shape Design[a]	7.00	5.10	1–16	10.47	2.60	7–16	2.49	31	.019	.111	0.87
Washer Design[a]	8.07	4.67	1–14	10.75	2.70	7–15	1.98	29	.058	.231	0.71
Parrots[a]	6.56	4.02	1–16	9.47	3.68	1–15	2.17	31	.019	.113	0.76
Boxes[a]	7.47	3.76	1–15	11.06	3.62	4–17	2.71	29	.006	.056	0.97
Digit and Letter Span[a]	7.19	3.12	1–14	9.76	2.33	5–14	2.70	31	.006	.056	0.94
Mixed Digit and Letter Span[a]	7.15	2.61	2–10	9.81	3.71	1–16	2.18	27	.019	.115	0.81
Shape Memory[a]	7.38	3.44	1–13	10.29	2.52	5–16	2.79	31	.004	**.049**	**0.97**
Rotated Shape Memory[a]	7.29	2.52	2–11	10.06	1.73	6–12	3.55	28	< .001	**.019**	**1.30**
Matrices: Completion[a]	7.44	3.81	3–18	10.71	3.20	4–17	2.67	31	.012	.092	0.93
Matrices: Odd One Out[a]	8.20	3.78	2–16	9.56	2.73	5–15	1.16	29	.257	.257	0.42
Naming Categories[a]	7.75	3.38	1–16	10.65	3.79	2–18	2.31	31	.014	.096	0.81
Naming Opposites[a]	7.73	3.56	1–13	11.00	2.07	6–14	3.15	29	.002	**.034**	**1.13**
Story Recall[a]	7.15	3.30	1–12	10.53	2.60	4–16	3.28	31	.001	**.029**	**1.14**
Picture Recall[a]	8.57	2.59	4–14	9.31	2.94	5–14	0.73	28	.236	.257	0.27

Variable	Autistic children $n = 17$			Non-autistic children $n = 17$			t	df	p	p_H	d
	M	SD	Range	M	SD	Range					
Executive functions composite score[b]	7.38	1.66	4–9	10.12	2.35	6–15	2.93	21	.004	**.047**	**1.28**
Listing Words[c]	7.00	2.28	4–11	10.14	3.25	5–16	2.72	23	.006	.058	1.09
Divided Attention[b]	7.06	2.01	4–10	10.13	2.93	5–14	2.77	22	.006	.055	1.17
Animal Colors[c]	6.30	3.33	1–12	8.80	3.80	3–15	1.69	23	.052	.210	0.69
Drawing Routes[b]	9.00	2.97	5–14	11.11	1.86	7–14	2.17	23	.020	.115	0.88

Note. Samples were matched for age, sex, and maternal education (as a proxy for socioeconomic status). p_H indicates p values adjusted with Hommel's (1988) correction. Significant results after accounting for multiple testing (Hommel correction) are presented in bold.

Table S4

Means, Standard Deviations, and t *tests of the Developmental Functions From the Intelligence and Development Scales–2 for Autistic and Non-Autistic Children (Aged 5-10 Years)*

Variable	Autistic children $n = 17$			Non-autistic children $n = 17$			*t*	*df*	*p*	p_H	*d*
	M	*SD*	Range	*M*	*SD*	Range					
Psychomotor skills composite score[b]	7.40	2.42	4–11	10.63	1.58	8–13	4.52	30	< .001	**.002**	**1.60**
Gross Motor Skills[a]	5.29	3.57	1–11	11.35	3.08	5–15	5.30	32	< .001	**< .001**	**1.82**
Fine Motor Skills[b]	8.25	3.10	3–13	10.50	2.73	4–15	2.18	30	.019	.113	0.77
Visuomotor Skills[b]	7.88	2.27	4–12	10.00	1.78	7–13	2.95	30	.003	**.041**	**1.04**
Social-emotional skills composite score[b]	7.70	3.02	2–11	10.67	1.99	6–13	3.38	32	.001	**.024**	**1.16**
Identifying Emotions[c]	7.71	4.27	1–12	10.24	2.68	4–12	2.07	32	.023	.117	0.71
Regulating Emotions[c]	7.53	3.61	1–13	10.94	2.86	5–15	3.06	32	.002	**.036**	**1.05**
Socially Competent Behavior[c]	7.88	3.24	1–12	10.82	2.90	5–15	2.79	32	.004	**.048**	**0.96**
Basic skills composite score[b]	6.99	2.24	2–10	9.91	2.21	5–13	3.19	24	.004	**.047**	**1.32**
Logical-Mathematical Reasoning[a]	5.80	3.05	1–13	9.82	3.36	3–15	3.53	30	.001	**.030**	**1.25**
Language Skills[b]	7.12	2.65	3–14	10.50	1.86	6–13	4.11	28	< .001	**.007**	**1.51**
Phoneme Analysis[a]	6.07	3.00	1–13	10.24	3.13	3–15	3.75	29	< .001	**.013**	**1.35**
Phoneme–Grapheme Correspondence[a]	9.07	4.20	1–15	10.00	2.18	5–13	0.79	29	.217	.257	0.29
Language Expressive[a]	7.08	2.96	1–14	10.41	2.55	5–15	3.31	28	.001	**.029**	**1.22**
Language Receptive[a]	6.71	3.34	2–14	11.35	2.37	7–16	4.52	29	< .001	**.002**	**1.63**
Reading[b]	5.86	3.44	1–12	9.78	2.80	5–14	3.32	26	.003	**.039**	**1.26**
Reading Words[c]	7.07	3.29	2–14	10.33	2.66	6–14	2.94	27	.007	.060	1.09
Reading Pseudo Words[c]	6.93	2.76	2–11	10.00	2.51	4–14	3.14	27	.004	**.047**	**1.17**
Text Comprehension[a]	5.50	5.35	1–15	10.92	2.64	5–14	3.14	22	.002	**.037**	**1.28**
Spelling[a]	5.71	1.98	3–8	9.87	2.72	5–15	3.60	20	.002	**.034**	**1.65**
Motivation and attitude composite score[b]	-	-	-	-	-	-	-	-	-	-	-
Conscientiousness[a]	-	-	-	-	-	-	-	-	-	-	-

Variable	Autistic children $n = 17$			Non-autistic children $n = 17$			t	df	p	p_H	d
	M	SD	Range	M	SD	Range					
Achievement Motivation[a]	-	-	-	-	-	-	-	-	-	-	-
Participation during testing, intelligence[a]	6.69	2.52	2–10	10.12	4.27	1–16	2.79	31	.005	.050	0.97
Participation during testing, executive functions[a]	8.09	1.30	7–10	10.43	3.44	7–16	2.13	23	.022	.115	0.86
Participation during testing, developmental functions[a]	6.59	2.98	1–10	10.57	3.59	5–16	3.38	29	.001	**.025**	**1.22**

Note. Samples were matched for age, sex, and maternal education (as a proxy for socioeconomic status). p_H indicates p values adjusted with Hommel's (1988) correction. Significant results after accounting for multiple testing (Hommel correction) are presented in bold. Conscientiousness and Achievement Motivation only for ages 11 to 20 years.

Table S5

Means, Standard Deviations, and t *tests of the Cognitive Functions From the Intelligence and Development Scales–2 for Autistic and Non-Autistic Adolescents (Aged 11-20 Years)*

Variable	Autistic adolescents $n = 26$			Non-autistic adolescents $n = 26$			*t*	*df*	*p*	p_H	*d*
	M	*SD*	Range	*M*	*SD*	Range					
Profile IQ[b]	96.64	16.91	55–131	95.81	13.91	61–121	-0.19	49	.576	.926	0.05
Full-Scale IQ[b]	98.80	17.60	55–129	96.31	12.75	63–116	-0.58	49	.718	.926	0.16
Screening IQ[b]	100.20	17.10	55–125	99.92	16.68	61–125	-0.06	49	.523	.926	0.02
Visual Processing[b]	103.92	13.93	77–123	102.69	13.22	84–129	-0.33	50	.745	.926	0.09
Processing Speed[b]	102.80	16.88	70–143	99.31	13.29	73–126	-0.82	49	.793	.926	0.23
Auditory Short-Term Memory[b]	95.58	17.01	55–139	98.81	10.84	74–121	0.82	50	.209	.926	0.23
Visuospatial Short-Term Memory[b]	93.00	13.24	64–118	95.08	10.41	78–118	0.62	49	.268	.926	0.17
Abstract Reasoning[b]	101.28	17.85	59–137	96.81	15.82	63–122	-0.95	49	.348	.926	0.27
Verbal Reasoning[b]	99.69	18.35	58–126	98.27	16.05	61–128	-0.30	50	.616	.926	0.08
Long-Term Memory[b]	89.52	15.71	55–113	91.19	17.08	58–137	0.36	49	.359	.926	0.10
Shape Design[a]	11.23	2.60	7–16	10.77	2.57	7–16	-0.64	50	.522	.926	0.18
Washer Design[a]	10.38	3.05	4–17	10.46	2.87	4–19	0.09	50	.926	.926	0.03
Parrots[a]	10.80	3.63	4–19	9.92	2.42	6–17	-1.02	49	.844	.926	0.29
Boxes[a]	10.36	2.46	5–16	10.00	3.20	2–15	-0.45	49	.672	.926	0.13
Digit and Letter Span[a]	10.15	3.57	1–18	10.08	2.10	5–14	-0.09	50	.538	.926	0.03
Mixed Digit and Letter Span[a]	9.15	3.29	1–18	10.46	2.50	6–17	1.61	50	.057	.926	0.45
Shape Memory[a]	9.08	2.52	4–14	8.62	2.04	5–13	-0.73	49	.764	.926	0.20
Rotated Shape Memory[a]	8.96	3.21	3–17	10.15	3.09	6–18	1.35	49	.091	.926	0.38
Matrices: Completion[a]	10.52	3.00	4–16	10.69	3.04	4–16	0.20	49	.840	.926	0.06
Matrices: Odd One Out[a]	10.64	3.62	3–18	8.88	3.06	2–14	-1.87	49	.067	.926	0.52
Naming Categories[a]	10.38	3.95	1–16	10.12	3.34	4–16	-0.27	50	.604	.926	0.07
Naming Opposites[a]	10.38	3.11	4–16	10.19	3.31	3–19	-0.22	50	.585	.926	0.06
Story Recall[a]	9.20	3.55	1–14	8.69	3.34	1–15	-0.53	49	.699	.926	0.15
Picture Recall[a]	7.77	2.80	3–13	8.65	3.57	3–18	0.99	50	.162	.926	0.28

Variable	Autistic adolescents $n = 26$			Non-autistic adolescents $n = 26$			t	df	p	p_H	d
	M	SD	Range	M	SD	Range					
Executive functions composite score[b]	9.31	2.17	6–13	9.84	1.76	6–14	0.96	48	.171	.926	0.27
Listing Words[c]	8.27	3.39	1–14	9.21	2.73	4–17	1.07	48	.144	.926	0.30
Divided Attention[b]	9.40	3.00	4–15	10.10	2.30	7–17	0.93	47	.180	.926	0.26
Animal Colors[c]	8.83	3.43	2–14	9.88	3.22	6–19	1.10	47	.138	.926	0.31
Drawing Routes[b]	10.29	2.39	5–15	10.02	2.63	5–15	-0.38	49	.648	.926	0.11

Note. Samples were matched for age, sex, and maternal education (as a proxy for socioeconomic status). p_H indicates p values adjusted with Hommel's (1988) correction. Please note that after this correction, none of the comparisons were significant.

Table S6

Means, Standard Deviations, and t *tests of the Developmental Functions From the Intelligence and Development Scales–2 for Autistic and Non-Autistic Adolescents (Aged 11-20 Years)*

Variable	Autistic adolescents $n = 26$			Non-autistic adolescents $n = 26$			t	df	p	p_H	d
	M	SD	Range	M	SD	Range					
Psychomotor skills composite score[b]	9.12	1.87	4–12	10.29	1.58	7–15	2.42	49	.010	.456	0.68
Gross Motor Skills[a]	-	-	-	-	-	-	-	-	-	-	-
Fine Motor Skills[b]	8.90	3.01	2–14	10.65	2.19	8–16	2.31	47	.013	.567	0.66
Visuomotor Skills[b]	9.35	1.55	6–13	10.02	1.74	7–13	1.46	49	.075	.926	0.41
Social-emotional skills composite score[b]	8.74	2.72	1–13	9.24	2.10	5–13	0.73	48	.235	.926	0.21
Identifying Emotions[c]	-	-	-	-	-	-	-	-	-	-	-
Regulating Emotions[c]	9.00	3.16	1–13	9.36	2.53	4–13	0.44	48	.329	.926	0.13
Socially Competent Behavior[c]	8.65	3.16	2–15	9.12	2.37	5–14	0.58	46	.281	.926	0.17
Basic skills composite score[b]	10.77	1.78	8–14	9.93	2.16	5–13	-1.49	48	.142	.926	0.42
Logical-Mathematical Reasoning[a]	10.96	3.61	1–17	10.52	2.90	4–16	-0.48	49	.633	.926	0.13
Language Skills[b]	-	-	-	-	-	-	-	-	-	-	-
Phoneme Analysis[a]	-	-	-	-	-	-	-	-	-	-	-
Phoneme–Grapheme Correspondence[a]	-	-	-	-	-	-	-	-	-	-	-
Language Expressive[a]	-	-	-	-	-	-	-	-	-	-	-
Language Receptive[a]	-	-	-	-	-	-	-	-	-	-	-
Reading[b]	10.40	1.84	8–15	9.58	2.19	5–13	-1.41	46	.167	.926	0.41
Reading Words[c]	10.67	2.76	5–16	8.96	2.41	5–13	-2.31	47	.025	.893	0.66
Reading Pseudo Words[c]	10.09	2.29	6–14	9.72	2.51	4–14	-0.53	46	.601	.926	0.15
Text Comprehension[a]	11.43	3.47	3–16	10.09	2.87	4–16	-1.43	44	.921	.926	0.42
Spelling[a]	9.95	2.80	6–15	9.75	2.67	4–14	-0.25	43	.806	.926	0.07

Variable	Autistic adolescents $n = 26$			Non-autistic adolescents $n = 26$			t	df	p	p_H	d
	M	SD	Range	M	SD	Range					
Motivation and attitude composite score[b]	10.56	3.24	6–17	10.65	2.78	6–19	0.11	46	.458	.926	0.03
Conscientiousness[a]	10.21	3.27	6–18	10.26	2.85	6–19	0.06	45	.477	.926	0.02
Achievement Motivation[a]	11.12	3.96	4–19	11.04	3.11	6–19	-0.07	47	.528	.926	0.02
Participation during testing, intelligence[a]	9.12	3.18	1–16	10.21	3.08	4–15	1.23	48	.112	.926	0.35
Participation during testing, executive functions[a]	9.04	3.16	1–16	10.00	2.56	4–14	1.14	46	.129	.926	0.33
Participation during testing, developmental functions[a]	9.46	3.06	1–16	10.71	2.71	5–16	1.52	48	.068	.926	0.43

Note. Samples were matched for age, sex, and maternal education (as a proxy for socioeconomic status). p_H indicates p values adjusted with Hommel's (1988) correction. Please note that after this correction, none of the comparisons were significant. Gross Motor Skills, Identifying Emotions, and Language Skills only for ages 5 to 10 years.
